# Supplementary material for: A newly detected bias in self-evaluation
Source: PLoS One. 2024 Feb 8;19(2):e0296383. doi: 10.1371/journal.pone.0296383 (PMC10852250; doi:10.1371/journal.pone.0296383)
Supplement: S1 File — (PDF) [file pone.0296383.s001.pdf]

A newly detected bias in self-evaluation: Supplementary Materials

1 Linear mixed effect models

Linear mixed effect models are relevant when the data are structured in different levels [1]. In particular, in our data, each participant generates 4 triples including self-evaluation, feedback and self evaluation change. The independence hypothesis between these values is not verified as they come from a single participant. The linear mixed effect method defines a linear approximation which takes the structure into account. It outputs fixed effects (or coefficients) that are the coefficients of a linear model, taking the structure into account, and random effects corresponding to a variance of these coefficients when the structure varies. We focus our analysis on the fixed effects which we expect to provide a sound linear approximation of the sensitivity to feedbacks. In practice, we use the `lmer` function from the `lme4` R package [2]. The typical function call is:

```
model = lmer(|deltaEval| ∼ eval + (eval | participant), data),
```

where `deltaEval` is the change of self-evaluation and `eval` the self-evaluation. This method is applicable only if there are more than two values of triples (self-evaluation, feedback and self evaluation change), otherwise, there is no meaningful second level. Therefore, we can use the `lmer` function only when including both positive and negative feedbacks and at least 3 time steps for each participant. This implies that the function cannot be used to compute the different measures of biases, as these measures require separating positive from negative feedbacks and there are only two time steps of each for each participant.

2 Complementary results

2.1 Sensitivity to positive or negative feedbacks

Table 1 shows the slopes c_p and c_n of the sensitivity to positive and negative feedbacks for different values of trust and different sets of time steps.

Overall, the slope c_n appears stronger and more significant than slope c_p. This suggests that the bias from sensitivity to feedbacks is mainly due to the sensitivity to negative feedbacks, especially for high trust. Moreover, for participants of high trust, $c_p - c_n$ the derivative of the self-enhancement bias is positive, suggesting that the self-enhancement bias increases with the self-evaluation. This is not true only for participants reporting low trust and $t \in (1:2)$.

2.2 Effect of anchor on bias from sensitivity

In the description of the experimental design, we explain that the probability of high anchor is twice the probability of low anchor because pilot experiments suggest that the decreasing sensitivity is more significant for sets of participants with a high anchor.

Trust	c_p			c_n		
	(1 : 2)	(1 : 3)	(1 : 4)	(1 : 2)	(1 : 3)	(1 : 4)
$[0, 10]$	-0.1^{**}	-0.05	0	-0.06 .	-0.11^{***}	-0.11^{***}
$[0, 6]$	-0.1^{*}	-0.05	0.01	-0.02	-0.07	-0.08^{*}
$[7, 10]$	-0.11 .	-0.05	-0.03	-0.16^{**}	-0.21^{***}	-0.18^{***}
$[8, 10]$	-0.15^{*}	-0.07	-0.06	-0.2^{**}	-0.22^{***}	-0.2^{***}
$[9, 10]$	-0.15	-0.06	-0.05	-0.25^{**}	-0.3^{***}	-0.25^{***}

*** : $p < 0.001$, ** : $p < 0.01$, * : $p < 0.05$, . : $p < 0.1$

Table 1. Slopes c_p and c_n of the sensitivity to positive and to negative feedbacks.

Slope of sensitivity c for low ($f_0 \leq 40$) and high ($f_0 \geq 60$) anchor

Trust	$f_0 \leq 40$		$f_0 \geq 60$	
	N	c	N	c
$[0, 10]$	680	-0.17^{**}	2056	-0.06
$[0, 6]$	444	-0.24^{***}	1212	-0.02
$[7, 10]$	236	-0.05	844	-0.13^{*}
$[8, 10]$	188	-0.04	646	-0.2^{**}
$[9, 10]$	104	-0.1	458	-0.2^{**}

*** : $p < 0.001$, ** : $p < 0.01$, * : $p < 0.05$, . : $p < 0.1$

Table 2. Slope of sensitivity c for $t \in (1:3)$ for low and high anchor. N is the size of the considered set.

Table 2 and 3 show respectively the slope of the sensitivity to feedbacks and the bias from sensitivity for sets distinguishing participants starting with low or high anchor and reporting different levels of trust. In sets of participants reporting high trust, the decreasing of sensitivity is significant and the bias from sensitivity is detected only when the anchor is high, which confirms the pilot studies. However, in the set of participants reporting low trust, the tendency is inverted: the slope of the sensitivity is significant and the bias is detected only when the anchor is low.

These results sets suggest that the sensitivity to the feedbacks is not linear as it decreases more significantly when the self-evaluation is in some ranges of values, like a logistic function for instance. Moreover, the range of self-evaluation for which the sensitivity decreases more significantly depends on the level of trust and possibly on the related level of involvement or attention.

Bias from sensitivity S' for low ($f_0 \leq 40$) and high ($f_0 \geq 60$) anchor

Trust	$f_0 \leq 40$			$f_0 \geq 60$		
	N	S' mean	S' std dev	N	S' mean	S' std dev
$[0, 10]$	1020	0.85	0.39	3084	0.59	0.24
$[0, 6]$	666	1.25	0.41	1818	0.31	0.32
$[7, 10]$	354	0.49	0.69	1266	1.13	0.36
$[8, 10]$	282	0.02	0.77	969	1.36	0.43
$[9, 10]$	156	1.02	1.04	687	1.59	0.48

Table 3. Bias from sensitivity S' for $t \in (1:3)$ for low and high anchor. N is the size of the considered set. The mean and standard deviation (std dev) are computed on 200 bootstrap samples.

Self-enhancement bias E for $t \in (1:2)$

Trust	crit.	Rank			Score		
		N	E mean	E std dev	N	E mean	E std dev
$[0, 10]$	All	1304	8.1	1.45	1432	-6.57	1.3
	$SE \leq 3$	664	8.05	2.11	678	-12.49	1.81
	$SE > 3$	640	8.09	1.98	754	-1.58	1.71
	Female	696	6.85	1.9	762	-11.74	1.78
	Male	608	9.27	2.13	670	-0.98	1.9
$[0, 6]$	All	828	8.54	1.68	828	-5.3	1.97
	$SE \leq 3$	440	9.09	2.63	384	-11.43	2.46
	$SE > 3$	388	7.61	2.57	444	-0.32	2.57
	Female	466	7.15	2.32	448	-10.98	2.5
	Male	362	10.19	2.79	380	1.42	2.87
$[7, 10]$	All	476	7.67	2.23	604	-8.98	1.7
	$SE \leq 3$	224	6	3.04	294	-14.64	2.58
	$SE > 3$	252	8.74	3.49	310	-3.76	2.38
	Female	230	7.2	3.34	314	-12.79	2.79
	Male	246	7.68	2.91	290	-4.93	2.51

Table 4. Self-enhancement bias E for different values of trust, scale, gender and self-esteem and $t \in (1:2)$. The values are the average (mean) and standard deviation (std dev) on 200 bootstrap samples.

2.3 Variations of self-enhancement and sensitivity biases with scale, gender and self-esteem for $t \in (1:2)$ and $t \in (1:4)$

Tables 4 and 5 show respectively variations of the measures of self-enhancement bias E and bias from sensitivity to feedbacks S', computed for $t \in (1:2)$ with scale, gender and self-esteem. Tables 8 and 9 show the same measures computed for $t \in (1:4)$. These tables should be compared to tables 6 and 7 of the main document which are analogous for $t \in (1:3)$. The main features are similar, with a higher standard deviation for time steps in $(1:2)$ because the sets are smaller.

2.4 Removing participants completing the questionnaire in less than 3 minutes

There are 14 participants who completed the questionnaire in less than 3 minutes. Each of these participant being associated with 4 triples $a_t^i, \delta_t^i, a_{t+1}^i$, hence 56 triples are removed from the data. Tables 10 and 11 show respectively the slope of the sensitivity to feedbacks and the mean and standard deviation of the bias from sensitivity computed on 200 bootstrap samples, when removing these participants from the data.

References

1. Nezlek JB. An introduction to Multilevel Modeling for Social and Personality Psychology. Social and Personality Psychology Compass. 2008;2(2):842–860.

2. Bates D, Machler M, Bolker B, Walker S. Fitting Linear Mixed-Effects Models Using lme4. Journal of Statistical Software. 2015;67(1):1–48. doi:10.18637/jss.v067.i01.

Theoretical sensitivity bias S' for $t \in (1:2)$

Trust	crit.	Rank			Score		
		N	S' mean	S' std dev	N	S' mean	S' std dev
$[0, 10]$	All	1304	0.21	0.28	1432	0.92	0.26
	$SE \leq 3$	664	-0.36	0.35	678	1.01	0.34
	$SE > 3$	640	0.83	0.39	754	0.97	0.4
	Female	696	0.03	0.37	762	1.14	0.35
	Male	608	0.34	0.44	670	0.65	0.41
$[0, 6]$	All	828	-0.1	0.35	828	0.98	0.34
	$SE \leq 3$	440	-0.64	0.48	384	1.01	0.43
	$SE > 3$	388	0.6	0.54	444	0.99	0.51
	Female	466	-0.21	0.43	448	1.34	0.47
	Male	362	0.06	0.55	380	0.37	0.49
$[7, 10]$	All	476	0.69	0.46	604	1.27	0.44
	$SE \leq 3$	224	0.02	0.71	294	1.26	0.64
	$SE > 3$	252	1.41	0.79	310	1.43	0.64
	Female	230	0.76	0.8	314	0.87	0.62
	Male	246	0.5	0.67	290	1.49	0.54

Table 5. Theoretical sensitivity bias S' for different values of trust, scale, gender and self-esteem and $t \in (1:2)$. The values are the average (mean) and standard deviation (std dev) on 200 bootstrap samples.

Self-enhancement bias E for $t \in (1:3)$

Trust	crit.	Rank			Score		
		N	E mean	E std dev	N	E mean	E std dev
$[0, 10]$	All	1956	8.86	1.23	2148	-6.18	1.09
	$SE \leq 3$	996	7.8	1.56	1017	-11.6	1.69
	$SE > 3$	960	10.27	1.59	1131	-1.34	1.68
	Female	1044	7.86	1.63	1143	-10.45	1.42
	Male	912	10.38	1.85	1005	-0.9	1.63
$[0, 6]$	All	1242	8.99	1.5	1242	-5.59	1.4
	$SE \leq 3$	660	7.43	2.14	576	-11.46	2.21
	$SE > 3$	582	10.82	2.19	666	-0.82	2.28
	Female	699	8.1	1.95	672	-10.89	2.13
	Male	543	10.36	2.28	570	0.51	2.34
$[7, 10]$	All	714	9.01	2.06	906	-7.19	1.64
	$SE \leq 3$	336	7.76	2.61	441	-11.98	2.5
	$SE > 3$	378	9.62	2.86	465	-2.65	2.06
	Female	345	7.1	2.89	471	-10.07	2.33
	Male	369	10.03	2.75	435	-4.1	2.32

Table 6. Enhancement bias E for different values of trust, scale, gender and self-esteem (SE) and $t \in (1:3)$. The values are the average (mean) and standard deviation (std dev) on 200 bootstrap samples.

Theoretical sensitivity bias S' for $t \in (1:3)$

Trust	crit.	Rank			Score		
		N	S' mean	S' std dev	N	S' mean	S' std dev
$[0,10]$	All	1956	0.29	0.25	2148	0.88	0.23
	$SE \leq 3$	996	-0.19	0.31	1017	1.09	0.25
	$SE > 3$	960	0.87	0.38	1131	0.68	0.35
	Female	1044	0.18	0.33	1143	1.2	0.3
	Male	912	0.36	0.36	1005	0.45	0.32
$[0,6]$	All	1242	0.02	0.27	1242	0.89	0.29
	$SE \leq 3$	660	-0.38	0.4	576	1.2	0.39
	$SE > 3$	582	0.44	0.48	666	0.57	0.43
	Female	699	-0.07	0.41	672	1.53	0.38
	Male	543	0.02	0.49	570	0.13	0.45
$[7,10]$	All	714	0.77	0.43	906	1.19	0.37
	$SE \leq 3$	336	0.03	0.55	441	1.03	0.53
	$SE > 3$	378	1.53	0.62	465	1.45	0.58
	Female	345	0.87	0.68	471	0.82	0.56
	Male	369	0.73	0.56	435	1.34	0.57

Table 7. Theoretical sensitivity bias S' for different values of trust, scale, gender and self-esteem (SE) and $t \in (1:3)$. The values are the average (mean) and standard deviation (std dev) on 200 bootstrap samples.

Self-enhancement bias E for $t \in (1:4)$

Trust	crit.	Rank			Score		
		N	E mean	E std dev	N	E mean	E std dev
$[0,10]$	All	2608	8.97	1.12	2864	-4.97	1.12
	$SE \leq 3$	1328	7.38	1.52	1356	-9.77	1.53
	$SE > 3$	1280	10.66	1.56	1508	-0.93	1.5
	Female	1392	7.76	1.57	1524	-8.46	1.35
	Male	1216	10.44	1.53	1340	-1	1.45
$[0,6]$	All	1656	9.48	1.41	1656	-5.1	1.57
	$SE \leq 3$	880	7.73	1.96	768	-9.72	2.37
	$SE > 3$	776	11.22	2.23	888	-0.58	2.04
	Female	932	8.07	1.92	896	-9.93	1.87
	Male	724	10.65	2.09	760	1	2.15
$[7,10]$	All	952	8.47	1.75	1208	-5.38	1.36
	$SE \leq 3$	448	6.67	2.71	588	-9.19	1.93
	$SE > 3$	504	10.07	2.56	620	-1.45	2
	Female	460	7.01	2.6	628	-6.61	2.2
	Male	492	9.66	2.24	580	-3.53	2.01

Table 8. Self-enhancement bias E for different values of trust, scale, gender and self-esteem and $t \in (1:4)$. The values are the average (mean) and standard deviation (std dev) on 200 bootstrap samples.

Theoretical sensitivity bias S' for $t \in (1:4)$

Trust	crit.	Rank			Score		
		N	S' mean	S' std dev	N	S' mean	S' std dev
$[0, 10]$	All	2608	0.13	0.23	2864	0.74	0.21
	$SE \leq 3$	1328	−0.25	0.34	1356	0.85	0.27
	$SE > 3$	1280	0.44	0.37	1508	0.62	0.37
	Female	1392	−0.01	0.32	1524	1.04	0.33
	Male	1216	0.23	0.31	1340	0.3	0.32
$[0, 6]$	All	1656	−0.12	0.28	1656	0.69	0.3
	$SE \leq 3$	880	−0.42	0.39	768	0.92	0.34
	$SE > 3$	776	0.15	0.46	888	0.36	0.47
	Female	932	−0.24	0.39	896	1.28	0.35
	Male	724	0	0.44	760	−0.06	0.44
$[7, 10]$	All	952	0.54	0.36	1208	1.08	0.34
	$SE \leq 3$	448	0.09	0.51	588	0.83	0.49
	$SE > 3$	504	1.01	0.49	620	1.46	0.55
	Female	460	0.47	0.66	628	0.93	0.5
	Male	492	0.51	0.42	580	1.23	0.44

Table 9. Theoretical sensitivity bias S' for different values of trust, scale, gender and self-esteem and $t \in (1:4)$. The values are the average (mean) and standard deviation (std dev) on 200 bootstrap samples.

Slope c of sensitivity to feedback for interview time greater than 3 minutes

Trust	$t \in (1:2)$		$t \in (1:3)$		$t \in (1:4)$	
	N	c	N	c	N	c
$[0, 10]$	2708	-0.08^{**}	4062	-0.08^{***}	5416	-0.06^{***}
$[0, 6]$	1636	-0.07^{*}	2454	-0.07^{*}	3272	-0.04 .
$[7; 10]$	1072	-0.12^{**}	1608	-0.12^{***}	2144	-0.11^{***}
$[8, 10]$	828	-0.16^{***}	1242	-0.14^{***}	1656	-0.13^{***}
$[9, 10]$	556	-0.18^{**}	834	-0.17^{***}	1112	-0.15^{***}

$*** : p < 0.001$, $** : p < 0.01$, $* : p < 0.05$, $. : p < 0.1$

Table 10. Slope c of sensitivity to feedback for interview time greater than 3 minutes.

Bias S' from sensitivity to feedbacks for interview time greater than 3 minutes

Trust	$t \in (1:2)$		$t \in (1:3)$	
	S' mean	S' std dev	S' mean	S' std dev
$[0, 10]$	0.59	0.19	0.58	0.16
$[0, 6]$	0.44	0.26	0.42	0.2
$[7, 10]$	0.95	0.34	0.95	0.3
$[8, 10]$	1.24	0.38	1.12	0.32
$[9, 10]$	1.52	0.54	1.3	0.39

Table 11. Bootstrap mean and standard deviation (std dev) of bias S' from sensitivity to feedbacks for interview time greater than 3 minutes, computed on 200 bootstrap samples.
