# Supplementary material for: A newly detected bias in self-evaluation
Source: PLoS One. 2024 Feb 8;19(2):e0296383. doi: 10.1371/journal.pone.0296383 (PMC10852250; doi:10.1371/journal.pone.0296383)
Supplement: S1 Appendix — The appendix explains how we computed linear mixed effect models for data structured in different levels [35] using the lme4 R package [34]. (PDF) [file pone.0296383.s002.pdf]

S1 Appendix. Linear mixed effect models

Linear mixed effect models are relevant when the data are structured in different levels. In particular, in our data, each participant generates 4 triples including self-evaluation, feedback and self evaluation change. The independence hypothesis between these values is not verified as they come from a single participant. The linear mixed effect method defines a linear approximation which takes the structure into account. It outputs fixed effects (or coefficients) that are the coefficients of a linear model, taking the structure into account, and random effects corresponding to a variance of these coefficients when the structure varies. We focus our analysis on the fixed effects which we expect to provide a sound linear approximation of the sensitivity to feedbacks. In practice, we use the `lmer` function from the `lme4` R package. The typical function call is:

```
model = lmer(|deltaEval| ∼ eval + (eval | participant), data),
```

where `deltaEval` is the change of self-evaluation and `eval` the self-evaluation. This method is applicable only if there are more than two values of triples (self-evaluation, feedback and self evaluation change), otherwise, there is no meaningful second level. Therefore, we can use the `lmer` function only when including both positive and negative feedbacks and at least 3 time steps for each participant. This implies that the function cannot be used to compute the different measures of biases, as these measures require separating positive from negative feedbacks and there are only two time steps of each for each participant.
