# Supplementary material for: A newly detected bias in self-evaluation
Source: PLoS One. 2024 Feb 8;19(2):e0296383. doi: 10.1371/journal.pone.0296383 (PMC10852250; doi:10.1371/journal.pone.0296383)
Supplement: S1 Table — The table shows the slopes cp and cn of the sensitivity to positive and negative feedbacks for different values of trust and different sets of time steps. Overall, the slope cn appears stronger and more significant than slope cp. This suggests that the bias from sensitivity to feedbacks is mainly due to the sensitivity to negative feedbacks, especially for high trust. Moreover, for participants of high trust, cp − cn the derivative of the self-enhancement bias is positive, suggesting that the self-enhancement bias increases with the self-evaluation. This is not true only for participants reporting low trust and t ∈ (1 : 2). (PDF) [file pone.0296383.s003.pdf]

S1 Table. Slopes c_p and c_n of the sensitivity to positive and to negative feedbacks.

Trust	c_p			c_n		
	(1 : 2)	(1 : 3)	(1 : 4)	(1 : 2)	(1 : 3)	(1 : 4)
$[0, 10]$	-0.1^{**}	-0.05	0	-0.06 .	-0.11^{***}	-0.11^{***}
$[0, 6]$	-0.1^{*}	-0.05	0.01	-0.02	-0.07	-0.08^{*}
$[7, 10]$	-0.11 .	-0.05	-0.03	-0.16^{**}	-0.21^{***}	-0.18^{***}
$[8, 10]$	-0.15^{*}	-0.07	-0.06	-0.2^{**}	-0.22^{***}	-0.2^{***}
$[9, 10]$	-0.15	-0.06	-0.05	-0.25^{**}	-0.3^{***}	-0.25^{***}

*** : $p < 0.001$, ** : $p < 0.01$, * : $p < 0.05$, . : $p < 0.1$
