# Supplementary material for: A newly detected bias in self-evaluation
Source: PLoS One. 2024 Feb 8;19(2):e0296383. doi: 10.1371/journal.pone.0296383 (PMC10852250; doi:10.1371/journal.pone.0296383)
Supplement: S2 Table — The table shows the slope of the sensitivity to feedbacks for sets distinguishing participants starting with low or high anchor and reporting different levels of trust. In sets of participants reporting high trust, the decreasing of sensitivity is significant only when the anchor is high, which confirms the pilot studies. However, in the set of participants reporting low trust, the tendency is inverted: the slope of the sensitivity is significant only when the anchor is low. These results suggest that the sensitivity to the feedbacks is not linear as it decreases more significantly when the self-evaluation is in some ranges of values, like a logistic function for instance. Moreover, the range of self-evaluation for which the sensitivity decreases more significantly depends on the level of trust and possibly on the related level of involvement or attention. (PDF) [file pone.0296383.s004.pdf]

S2 Table. Slope of sensitivity c for $t \in (1:3)$ for low and high anchor. N is the size of the considered set.

Slope of sensitivity c for low ($f_0 \leq 40$) and high ($f_0 \geq 60$) anchor

Trust	$f_0 \leq 40$		$f_0 \geq 60$	
	N	c	N	c
$[0, 10]$	680	-0.17^{**}	2056	-0.06
$[0, 6]$	444	-0.24^{***}	1212	-0.02
$[7, 10]$	236	-0.05	844	-0.13^{*}
$[8, 10]$	188	-0.04	646	-0.2^{**}
$[9, 10]$	104	-0.1	458	-0.2^{**}

*** : $p < 0.001$, ** : $p < 0.01$, * : $p < 0.05$, . : $p < 0.1$
