# Supplementary material for: A newly detected bias in self-evaluation
Source: PLoS One. 2024 Feb 8;19(2):e0296383. doi: 10.1371/journal.pone.0296383 (PMC10852250; doi:10.1371/journal.pone.0296383)
Supplement: S3 Table — The table shows the bias from sensitivity for sets distinguishing participants starting with low or high anchor and reporting different levels of trust. In sets of participants reporting high trust, the bias from sensitivity is significant only when the anchor is high, which confirms the pilot studies. However, in the set of participants reporting low trust, the tendency is inverted: the bias from sensitivity is significant only when the anchor is low. (PDF) [file pone.0296383.s005.pdf]

S3 Table. Bias from sensitivity $S'$ for $t \in (1:3)$ for low and high anchor ($f_0$). $N$ is the size of the considered set. The mean and standard deviation (std dev) are computed on 200 bootstrap samples.

| Trust | $f_0 \leq 40$ | | | $f_0 \geq 60$ | | |
|---|---|---|---|---|---|---|
| | $N$ | $S'$ mean | $S'$ std dev | $N$ | $S'$ mean | $S'$ std dev |
| $[0, 10]$ | 1020 | 0.85 | 0.39 | 3084 | 0.59 | 0.24 |
| $[0, 6]$ | 666 | 1.25 | 0.41 | 1818 | 0.31 | 0.32 |
| $[7, 10]$ | 354 | 0.49 | 0.69 | 1266 | 1.13 | 0.36 |
| $[8, 10]$ | 282 | 0.02 | 0.77 | 969 | 1.36 | 0.43 |
| $[9, 10]$ | 156 | 1.02 | 1.04 | 687 | 1.59 | 0.48 |
