# Supplementary material for: A newly detected bias in self-evaluation
Source: PLoS One. 2024 Feb 8;19(2):e0296383. doi: 10.1371/journal.pone.0296383 (PMC10852250; doi:10.1371/journal.pone.0296383)
Supplement: S4 Table — The table shows the variations of the measures of self-enhancement bias E computed for t ∈ (1 : 2) with scale, gender and self-esteem. The main features are similar to the ones of the same table for t ∈ (1 : 2) shown in the main text, with a higher standard deviation for time steps in (1 : 2) because the sets are smaller. (PDF) [file pone.0296383.s006.pdf]

S4 Table. Self-enhancement bias E for different values of trust, scale, gender and self-esteem and $t \in (1:2)$. The values are the average (mean) and standard deviation (std dev) on 200 bootstrap samples.

Trust	crit.	Rank			Score		
		N	E mean	E std dev	N	E mean	E std dev
$[0, 10]$	All	1304	8.1	1.45	1432	-6.57	1.3
	$SE \leq 3$	664	8.05	2.11	678	-12.49	1.81
	$SE > 3$	640	8.09	1.98	754	-1.58	1.71
	Female	696	6.85	1.9	762	-11.74	1.78
	Male	608	9.27	2.13	670	-0.98	1.9
$[0, 6]$	All	828	8.54	1.68	828	-5.3	1.97
	$SE \leq 3$	440	9.09	2.63	384	-11.43	2.46
	$SE > 3$	388	7.61	2.57	444	-0.32	2.57
	Female	466	7.15	2.32	448	-10.98	2.5
	Male	362	10.19	2.79	380	1.42	2.87
$[7, 10]$	All	476	7.67	2.23	604	-8.98	1.7
	$SE \leq 3$	224	6	3.04	294	-14.64	2.58
	$SE > 3$	252	8.74	3.49	310	-3.76	2.38
	Female	230	7.2	3.34	314	-12.79	2.79
	Male	246	7.68	2.91	290	-4.93	2.51
