# Supplementary material for: A newly detected bias in self-evaluation
Source: PLoS One. 2024 Feb 8;19(2):e0296383. doi: 10.1371/journal.pone.0296383 (PMC10852250; doi:10.1371/journal.pone.0296383)
Supplement: S5 Table — The table shows the variations of the measures theoretical sensitivity bias S′ for t ∈ (1 : 2) with scale, gender and self-esteem. The main features are similar to the ones of the same table for t ∈ (1 : 3) and t ∈ (1 : 4), with a higher standard deviation for time steps in (1 : 2) because the sets are smaller. (PDF) [file pone.0296383.s007.pdf]

S5 Table. Theoretical sensitivity bias $S'$ for different values of trust, scale, gender and self-esteem and $t \in (1:2)$. The values are the average (mean) and standard deviation (std dev) on 200 bootstrap samples.

| Trust | crit. | Rank | | | Score | | |
|---|---|---|---|---|---|---|---|
| | | $N$ | $S'$ mean | $S'$ std dev | $N$ | $S'$ mean | $S'$ std dev |
| [0, 10] | All | 1304 | 0.21 | 0.28 | 1432 | 0.92 | 0.26 |
| | $SE \leq 3$ | 664 | −0.36 | 0.35 | 678 | 1.01 | 0.34 |
| | $SE > 3$ | 640 | 0.83 | 0.39 | 754 | 0.97 | 0.4 |
| | Female | 696 | 0.03 | 0.37 | 762 | 1.14 | 0.35 |
| | Male | 608 | 0.34 | 0.44 | 670 | 0.65 | 0.41 |
| [0, 6] | All | 828 | −0.1 | 0.35 | 828 | 0.98 | 0.34 |
| | $SE \leq 3$ | 440 | −0.64 | 0.48 | 384 | 1.01 | 0.43 |
| | $SE > 3$ | 388 | 0.6 | 0.54 | 444 | 0.99 | 0.51 |
| | Female | 466 | −0.21 | 0.43 | 448 | 1.34 | 0.47 |
| | Male | 362 | 0.06 | 0.55 | 380 | 0.37 | 0.49 |
| [7, 10] | All | 476 | 0.69 | 0.46 | 604 | 1.27 | 0.44 |
| | $SE \leq 3$ | 224 | 0.02 | 0.71 | 294 | 1.26 | 0.64 |
| | $SE > 3$ | 252 | 1.41 | 0.79 | 310 | 1.43 | 0.64 |
| | Female | 230 | 0.76 | 0.8 | 314 | 0.87 | 0.62 |
| | Male | 246 | 0.5 | 0.67 | 290 | 1.49 | 0.54 |
