# Supplementary material for: A newly detected bias in self-evaluation
Source: PLoS One. 2024 Feb 8;19(2):e0296383. doi: 10.1371/journal.pone.0296383 (PMC10852250; doi:10.1371/journal.pone.0296383)
Supplement: S6 Table — The table shows the variations of the measures of self-enhancement bias E computed for t ∈ (1 : 3) with scale, gender and self-esteem. (PDF) [file pone.0296383.s008.pdf]

S6 Table. Enhancement bias $E$ for different values of trust, scale, gender and self-esteem (SE) and $t \in (1:3)$. The values are the average (mean) and standard deviation (std dev) on 200 bootstrap samples.

| Trust | crit. | Rank | | | Score | | |
|---|---|---|---|---|---|---|---|
| | | $N$ | $E$ mean | $E$ std dev | $N$ | $E$ mean | $E$ std dev |
| $[0, 10]$ | All | 1956 | 8.86 | 1.23 | 2148 | $-6.18$ | 1.09 |
| | $SE \leq 3$ | 996 | 7.8 | 1.56 | 1017 | $-11.6$ | 1.69 |
| | $SE > 3$ | 960 | 10.27 | 1.59 | 1131 | $-1.34$ | 1.68 |
| | Female | 1044 | 7.86 | 1.63 | 1143 | $-10.45$ | 1.42 |
| | Male | 912 | 10.38 | 1.85 | 1005 | $-0.9$ | 1.63 |
| $[0, 6]$ | All | 1242 | 8.99 | 1.5 | 1242 | $-5.59$ | 1.4 |
| | $SE \leq 3$ | 660 | 7.43 | 2.14 | 576 | $-11.46$ | 2.21 |
| | $SE > 3$ | 582 | 10.82 | 2.19 | 666 | $-0.82$ | 2.28 |
| | Female | 699 | 8.1 | 1.95 | 672 | $-10.89$ | 2.13 |
| | Male | 543 | 10.36 | 2.28 | 570 | 0.51 | 2.34 |
| $[7, 10]$ | All | 714 | 9.01 | 2.06 | 906 | $-7.19$ | 1.64 |
| | $SE \leq 3$ | 336 | 7.76 | 2.61 | 441 | $-11.98$ | 2.5 |
| | $SE > 3$ | 378 | 9.62 | 2.86 | 465 | $-2.65$ | 2.06 |
| | Female | 345 | 7.1 | 2.89 | 471 | $-10.07$ | 2.33 |
| | Male | 369 | 10.03 | 2.75 | 435 | $-4.1$ | 2.32 |
