# Supplementary material for: A newly detected bias in self-evaluation
Source: PLoS One. 2024 Feb 8;19(2):e0296383. doi: 10.1371/journal.pone.0296383 (PMC10852250; doi:10.1371/journal.pone.0296383)
Supplement: S7 Table — The table shows the variations of the measures theoretical sensitivity bias S′ for t ∈ (1 : 3) with scale, gender and self-esteem. (PDF) [file pone.0296383.s009.pdf]

S7 Table. Theoretical sensitivity bias S' for different values of trust, scale, gender and self-esteem (SE) and $t \in (1:3)$. The values are the average (mean) and standard deviation (std dev) on 200 bootstrap samples.

Trust	crit.	Rank			Score		
		N	S' mean	S' std dev	N	S' mean	S' std dev
$[0, 10]$	All	1956	0.29	0.25	2148	0.88	0.23
	$SE \leq 3$	996	−0.19	0.31	1017	1.09	0.25
	$SE > 3$	960	0.87	0.38	1131	0.68	0.35
	Female	1044	0.18	0.33	1143	1.2	0.3
	Male	912	0.36	0.36	1005	0.45	0.32
$[0, 6]$	All	1242	0.02	0.27	1242	0.89	0.29
	$SE \leq 3$	660	−0.38	0.4	576	1.2	0.39
	$SE > 3$	582	0.44	0.48	666	0.57	0.43
	Female	699	−0.07	0.41	672	1.53	0.38
	Male	543	0.02	0.49	570	0.13	0.45
$[7, 10]$	All	714	0.77	0.43	906	1.19	0.37
	$SE \leq 3$	336	0.03	0.55	441	1.03	0.53
	$SE > 3$	378	1.53	0.62	465	1.45	0.58
	Female	345	0.87	0.68	471	0.82	0.56
	Male	369	0.73	0.56	435	1.34	0.57
