# Supplementary material for: A newly detected bias in self-evaluation
Source: PLoS One. 2024 Feb 8;19(2):e0296383. doi: 10.1371/journal.pone.0296383 (PMC10852250; doi:10.1371/journal.pone.0296383)
Supplement: S8 Table — The table shows the variations of the measures of self-enhancement bias E computed for t ∈ (1 : 3) with scale, gender and self-esteem. (PDF) [file pone.0296383.s010.pdf]

S8 Table. Self-enhancement bias E for different values of trust, scale, gender and self-esteem and $t \in (1:4)$. The values are the average (mean) and standard deviation (std dev) on 200 bootstrap samples.

Trust	crit.	Rank			Score		
		N	E mean	E std dev	N	E mean	E std dev
$[0, 10]$	All	2608	8.97	1.12	2864	-4.97	1.12
	$SE \leq 3$	1328	7.38	1.52	1356	-9.77	1.53
	$SE > 3$	1280	10.66	1.56	1508	-0.93	1.5
	Female	1392	7.76	1.57	1524	-8.46	1.35
	Male	1216	10.44	1.53	1340	-1	1.45
$[0, 6]$	All	1656	9.48	1.41	1656	-5.1	1.57
	$SE \leq 3$	880	7.73	1.96	768	-9.72	2.37
	$SE > 3$	776	11.22	2.23	888	-0.58	2.04
	Female	932	8.07	1.92	896	-9.93	1.87
	Male	724	10.65	2.09	760	1	2.15
$[7, 10]$	All	952	8.47	1.75	1208	-5.38	1.36
	$SE \leq 3$	448	6.67	2.71	588	-9.19	1.93
	$SE > 3$	504	10.07	2.56	620	-1.45	2
	Female	460	7.01	2.6	628	-6.61	2.2
	Male	492	9.66	2.24	580	-3.53	2.01
