# Supplementary material for: A newly detected bias in self-evaluation
Source: PLoS One. 2024 Feb 8;19(2):e0296383. doi: 10.1371/journal.pone.0296383 (PMC10852250; doi:10.1371/journal.pone.0296383)
Supplement: S9 Table — The table shows the variations of the measures theoretical sensitivity bias S′ for t ∈ (1 : 3) with scale, gender and self-esteem. (PDF) [file pone.0296383.s011.pdf]

S9 Table. Theoretical sensitivity bias S' for different values of trust, scale, gender and self-esteem and $t \in (1:4)$. The values are the average (mean) and standard deviation (std dev) on 200 bootstrap samples.

Trust	crit.	Rank			Score		
		N	S' mean	S' std dev	N	S' mean	S' std dev
$[0, 10]$	All	2608	0.13	0.23	2864	0.74	0.21
	$SE \leq 3$	1328	-0.25	0.34	1356	0.85	0.27
	$SE > 3$	1280	0.44	0.37	1508	0.62	0.37
	Female	1392	-0.01	0.32	1524	1.04	0.33
	Male	1216	0.23	0.31	1340	0.3	0.32
$[0, 6]$	All	1656	-0.12	0.28	1656	0.69	0.3
	$SE \leq 3$	880	-0.42	0.39	768	0.92	0.34
	$SE > 3$	776	0.15	0.46	888	0.36	0.47
	Female	932	-0.24	0.39	896	1.28	0.35
	Male	724	0	0.44	760	-0.06	0.44
$[7, 10]$	All	952	0.54	0.36	1208	1.08	0.34
	$SE \leq 3$	448	0.09	0.51	588	0.83	0.49
	$SE > 3$	504	1.01	0.49	620	1.46	0.55
	Female	460	0.47	0.66	628	0.93	0.5
	Male	492	0.51	0.42	580	1.23	0.44
