# Supplementary material for: A newly detected bias in self-evaluation
Source: PLoS One. 2024 Feb 8;19(2):e0296383. doi: 10.1371/journal.pone.0296383 (PMC10852250; doi:10.1371/journal.pone.0296383)
Supplement: S10 Table — The table shows the slope of the sensitivity to feedbacks, when removing the 14 participants who took less than 3 minutes to fill the questionnaire from the data (56 triples ati,δti,at+1i removed from the data). (PDF) [file pone.0296383.s012.pdf]

S10 Table. Slope c of sensitivity to feedback for interview time greater than 3 minutes.

Trust	$t \in (1:2)$		$t \in (1:3)$		$t \in (1:4)$	
	N	c	N	c	N	c
$[0, 10]$	2708	-0.08^{**}	4062	-0.08^{***}	5416	-0.06^{***}
$[0, 6]$	1636	-0.07^{*}	2454	-0.07^{*}	3272	-0.04 .
$[7; 10]$	1072	-0.12^{**}	1608	-0.12^{***}	2144	-0.11^{***}
$[8, 10]$	828	-0.16^{***}	1242	-0.14^{***}	1656	-0.13^{***}
$[9, 10]$	556	-0.18^{**}	834	-0.17^{***}	1112	-0.15^{***}

*** : $p < 0.001$, ** : $p < 0.01$, * : $p < 0.05$, . : $p < 0.1$
