# Supplementary material for: A newly detected bias in self-evaluation
Source: PLoS One. 2024 Feb 8;19(2):e0296383. doi: 10.1371/journal.pone.0296383 (PMC10852250; doi:10.1371/journal.pone.0296383)
Supplement: S11 Table — The table shows the slope of the sensitivity to feedbacks and the mean and standard deviation of the bias from sensitivity computed on 200 bootstrap samples, when removing the 14 participants who took less than 3 minutes to fill the questionnaire from the data (56 triples ati,δti,at+1i removed from the data). (PDF) [file pone.0296383.s013.pdf]

S11 Table. Bootstrap mean and standard deviation (std dev) of bias S' from sensitivity to feedbacks for interview time greater than 3 minutes, computed on 200 bootstrap samples.

Trust	$t \in (1:2)$		$t \in (1:3)$	
	S' mean	S' std dev	S' mean	S' std dev
$[0, 10]$	0.59	0.19	0.58	0.16
$[0, 6]$	0.44	0.26	0.42	0.2
$[7, 10]$	0.95	0.34	0.95	0.3
$[8, 10]$	1.24	0.38	1.12	0.32
$[9, 10]$	1.52	0.54	1.3	0.39
