# Supplementary material for: A newly detected bias in self-evaluation
Source: PLoS One. 2024 Feb 8;19(2):e0296383. doi: 10.1371/journal.pone.0296383 (PMC10852250; doi:10.1371/journal.pone.0296383)
Supplement: Attachment — Submitted filename: answerToReviewers.pdf [file pone.0296383.s014.pdf]

Dear Editor,

We thank you and the revisers for your very valuable feedback on our paper.

Please find below the detailed explanations of the changes we made, answering point by point your requirements and the remarks or criticism from the reviewers. Our answers are in blue and preceded by the prompt “->”.

The new version of the paper is very different from the previous one and it may be useful to present briefly the main changes: Following your suggestions, we simplified the result section, keeping only the most important results and putting all the others and complementary ones in a supplementary materials document. Moreover, we deeply rewrote the introduction and the discussion.

In addition, we would like to mention two changes that were not requested:

- We cite a new reference: (Muller-Piznzler et al, 2019, ref 19) reporting an experiment which shares several features with ours. This experiment uses only the scale that we call score and its results show a negative bias, like ours. This paper is therefore an important reference that we use in the introduction, the experimental design and the discussion.
- We computed bootstrap standard deviations on the measures of the biases. They provide an assessment of the significance of these measures and of their differences.

Overall, we think that this version is significantly stronger than the previous one, and we thank you again for your help.

Best regards,

Editor:
In line with reviewers' comments, your major revision will require:

1. More detailed theoretical background (see helpful comments by reviewer #2) and introduction, including the rationale for all included variables in your models.

➔ We have rewritten a large part of the introduction. The new version includes a new paragraph of background, which uses most of the references suggested by reviewer 2. We also tried to explain better the rationale of our approach, distinguishing the model of self-evaluation change in reaction to a feedback and the two biases that are defined from it.

2. Restating of the research goals so that they correspond more closely to your analyses.

➔ We restated the research goals as follows:

In this paper, our primary objective is to bring experimental evidence supporting two main hypotheses:

1. People show an average decreasing sensitivity to feedbacks, when their self-evaluation increases;
2. This induces a specific positive bias in self-evaluation that is added to self-enhancement (or self-derogation) bias.

Our secondary objective is to observe how both sensitivity and self-enhancement biases are modulated by variables such as evaluation scale, gender and self-esteem. 3

.A different, more suitable analytical approach (multilevel modelling that aligns with the research design)

- ➔ We completely rewrote the results section. We followed more closely the distinction between our two objectives. We put the hierarchical linear models in the Supplementary Materials, as they do not directly contribute to our main results.

4. More clarity and detailed report on data analysis e.g., model specifications, diagnostics. Also making your code available - I suggest providing a link to a GitHub account with the code - would help clarify some of concerns and questions of the reviewers regarding the analyses you have done. Finally, the revision will require including some additional tables and figures - possibly as Supplementary Materials (see reviewer #2 comments).

- ➔ In the new version of the result section, we tried to explicit more clearly the connections with the method section. We made the R-code accessible on GitHub and we added many supplementary tables in the Supplementary Materials.

Reviewer #1: I reviewed a previous version of this paper. In this revision the authors have addressed some of the previous concerns, for example by offering a rationale for why higher self-evaluations for a highly specific task might be associated with lower responsiveness to feedback on performance. The paper's underlying idea remains interesting, a new process (different from conventional self-enhancement) that can lead to exaggerated self-evaluations. A number of concerns remain, however.

In the most general terms, the main problem is a mismatch between the paper's stated goal, testing a theoretical prediction derived from earlier modeling work in a human-subjects experiment, and what is actually done, which has a strongly exploratory flavor.

- ➔ We restated the goals as follows:

In this paper, our primary objective is to bring experimental evidence supporting two main hypotheses:

1. People show an average decreasing sensitivity to feedbacks, when their self-evaluation increases;
2. This induces a specific positive bias in self-evaluation that is added to self-enhancement (or self-derogation) bias.

Our secondary objective is to observe how both sensitivity and self-enhancement biases are modulated by variables such as evaluation scale, gender and self-esteem.

There are also important areas of unclarity, for example about the details of the analyses.

The paper's stated goal would best be met by an analysis that (a) omitted participants who reported low levels of trust in the manipulation – because such participants are not relevant for testing the hypothesis

- ➔ Our main hypotheses are also confirmed for the participants who reported low levels of trust (though the results for participants reporting high levels of trust are more significant). We tried to make this point clearer in this new version.

– and (b) used all the remaining participants in a multilevel analysis that accounts for nonindependence of responses from the same individual. As far as I can tell, no such analysis is reported.

- ➔ We rewrote the section about the HLM, which precisely investigates possible non-independencies. We tried to clarify that the linear models derived for single participants have non-significant coefficients in more than 90 % of the cases (first level of HLM 1). Hence the structure at the individual level does not seem reliable. The results of model HLM2 suggest that there is no significant temporal tendency either. We conclude that it is statistically correct to derive linear models mixing participants and time steps. We put this section in the Supplementary Materials though it is an important methodological point, because we think that it does not directly contribute to our objectives.

Instead, the analyses mostly retain the inappropriate low-trust participants, and slice and dice the data into many subsets for separate analyses (for example, only the first two or the first 3 responses from the 4 trials; males versus females; high versus low self-esteem; two different scoring methods), as seen in Tables 4 through 10. The problem is that multiple low-powered analyses (low-powered because they use only subsets of the data) give highly variable results, and some will pop up as significant by chance alone without these results being meaningful. A better approach would be to conduct a single analysis using all high-trust participants to test the overall hypothesis, and perhaps follow that up with exploratory analyses to examine trends by gender or self-esteem, etc.

- ➔ The reviewer seems to have missed that the results about all participants of high trust were actually reported in tables 4 to 10. In the new version, we separated the results about our main hypotheses that are based on all data considering only different levels of trust, from the observation of variations of the biases depending on self-esteem, gender or scale. Moreover, we computed a standard deviation on bootstrap samples, which provides an indication on the significance of the measures of biases.

A related concern is that the potential moderator variables that are examined (scoring method, gender, self-esteem and so on) are not theoretically motivated – there is no conceptual discussion of why these variables should influence the magnitude of the effect. In fact, one atheoretical factor (rank versus score) ends up reversing the predicted effect (top of p. 24), which can be given only a speculative interpretation.

➔ The reviewer is referring to the strong self-derogation found when the participants self-evaluate by score. In the new version, we relate this result to (Muller-Piznzler et al, 2019, ref 19) which reports an experiment sharing several features with ours, in particular this scale (score) and which also finds a significant negative bias. Indeed, Muller-Piznzler et al, propose a theoretical explanation of their result that we discuss, in the light of our other results when using the rank scale (which Muller-Piznzler et al, did not test). Therefore, we think that these results are better related to the literature in the new version.

The whole paper comes across as a large-scale exercise in data-fishing rather than as a focused test of an interesting a priori hypothesis.

➔ We hope that with the new formulation of the objectives and the new presentation of the results, both distinguishing the main objective of the paper (testing our main hypotheses) and secondary objectives (observing how the biases vary when scale, self-esteem and gender vary), this reviewer's impression could change.

There is still a concern that participants were required to report a new self-rating between their former rating and the new feedback – in other words, the program forced participants to modify their self-evaluations in the way described by the mathematical model. This may partially account for any agreement between the experimental data and the theoretical predictions.

➔ The reviewer may have missed our answer to this criticism. In the new version, it is included in the section entitled "overview of the experiment" and we reproduce it below for the convenience of the reviewer. Without any feedback from the reviewer about this answer, we left it unchanged.

- The participants are asked their expected evaluation a_2 in group G_2. They are requested to express this self-evaluation between their previous expectation a_1 and the feedback f_1 that they just received. Doing this, we impose that the sensitivity to the feedback is between 0 and 1 for each observation. The literature (e.g. [23]) and several pilot experiments that we made (not-reported here) suggest that this assumption holds in a large majority of cases. Therefore, the constraint on the self-evaluation is primarily a means to limit the noise in the results. Moreover, the way in which the sensitivity changes within the bounds, when the self-evaluation varies, is our main subject of investigation and it is not constrained.

The exact details of the multilevel (hierarchical) analyses HLM1 and HLM2 not specified on p. 9. The model equation or model specification in lmer syntax for R (for example) should be provided.

➔ We added more details about HLM1 and HLM2 (see our previous answer). Moreover, the R code of all the treatments is now available on github; https://github.com/guillaumeDeffuant/sensitivityBias/

Reviewer #2: The authors present a very interesting paper dedicated to investigate the interplay between self-esteem, sensitivity to feedback and self-enhancement. They propose to research this important topic by an experimental study in which participants assess an area of irregular geometric shapes and then self-assess their performance. In addition, participants are

given experimentally manipulated feedback, which is either positive or negative. Overall, this is a very interesting study with potentially novel contribution.

➔ We thank the reviewer for this appreciation.

However, reading the article I could not shrug off the impression that the paper resembles more a technical report than a full-blown research paper. This is mainly due to a very cursory introduction, in which, in my opinion, the theses of the research are not sufficiently introduced and explained. The authors refer to agent-model studies that have established a relation between the level of self-assessment and sensitivity to feedback, pointing to a decreased sensitivity to feedback as one of the sources of self-enhancement. This is a very interesting research, but I would like to see more theoretical elaboration on it as simple references to some previous studies do not suffice. The literature on sources and mechanisms of self-enhancement is vast, so I recommend introducing the ideas of the research in the context of a proper literature review. The list of exemplary publications to which the authors can refer contains, i.a.:

➔ We warmly thank the reviewer for providing these references. We tried our best to use them in the new version of the introduction.

The authors should also comment on the role of the domain in which participants self-assess and receive feedback with regard to its relevance/importance and agency/communion values.

➔ We included these points in the discussion about our results on the gender differences in the self-enhancement bias.

Besides the request to write a much broader literature review, I would also like to rise some minor issues: Anchor: Why 1/3 of the participants were given low anchor and 2/3 high anchor and not 50:50? Was that done solely to have more data for respondents with higher anchor?

➔ Yes, as mentioned in the paper, we take more participants with high anchor because in pilot studies (with less data) we found that our hypotheses were confirmed only for high anchor. In the new version, the effect of anchor is mentioned in the result section as follows:

> Moreover, in order to keep a reasonable number of tables in the main text, the variations of the sensitivity bias with the anchor, which seem to us more peripheral, are available in the Supplementary Materials (tables 6 and 7).

These tables confirm the results of the pilot studies in sets of participants of high level of trust. The results are different in sets of participants of low level of trust (see the comments on the tables in Supplementary Materials)

Research design: In general, authors should re-write that part of the paper and explain their experimental design in clear and short terms. If I am not mistaken, the design is in fact close to 6 (type of feedback sequence) x 2 (type of feedback: score or rank), so authors should present it more clearly in terms understandable to interdisciplinary readership.

➔ We modified the presentation of the experiment. We added an overview schema. The new description of the experimental design is as follows:

The experimental design includes the following conditions:

- Low anchor (randomly chosen in [15, 40]) vs high anchor (randomly chosen in [60, 85]).
- Six possible series of feedbacks (shown on table 1);
- Evaluation by rank vs evaluation by score:
  - The rank is the number of persons (within the considered group of 100 persons) who perform better than the participant. 1 is the best rank, 101 is the worst;
  - The score is the number of persons in the group who perform worse than the participant. 100 is the best score, 0 is the worst. This is the scale used in [19].

In total there are 24 different conditions: 2 (anchor) x 6 (feedback sequences) x 2 (scale). All the conditions have the same probability, except the high anchor, which has a higher probability than the low anchor ($\frac{2}{3}$ vs $\frac{1}{3}$). Indeed, a pilot experiment suggested that sensitivity to feedbacks decreases only when the anchor is high. Therefore it appeared important to collect more data in these conditions.

In [19] the evaluation is made by score only. However, it is important in our experiment to check that a possible decreasing sensitivity is independent from the chosen scale.

I also think that the paper's clarity would benefit from commenting and explaining more about the regression equations used to analyse the results, including their hierarchical character. I think some graphs or diagrams could help here, otherwise the reader is a bit lost in the overflow of equations and tables.

➔ We rewrote completely the result section. We repeated the equation of the sensitivity and referred more precisely to the method section for the computation of the biases. We limited the number of tables in the main document. In particular, we put the work on the hierarchical models in the Supplementary Materials, as they do not contribute directly to confirming our main hypotheses.

Slopes and Intercepts: I would appreciate if the authors could possibly present all the regression coefficients in a clear way before presenting the numerical results. Otherwise, it is difficult to follow what all those different slopes and intercepts mean.

➔ We believe that this problem came mainly from the HLMs. We rewrote completely their presentation in the Supplementary Materials, trying to clarify the meaning of each variable.

Tables and Figures layout: The tables could be a bit more readable and more conforming to standards of communication, e.g., explaining what does the "*" stand for (I assume it's for the p-levels but it is not explained in the paper).

➔ Yes, these codes indicate the usual p-levels. We specified them in the new version.

Also, the tables and figures lack titles.

➔ We added titles above the tables.

Self-disparagement: I would also suggest replacing the term “self-disparagement” with something more often used in the self-motives literature, e.g. terms “self-effacement”, “self-derogation”, or “self-diminishment” (e.g. Kiu, Chiu & Zou, 2010). “Self-disparagement” seems to be used in other contexts, e.g. depression or humour research. In my opinion, we should avoid multiplying terms and should stick to the already many terms used in the self-motives field.

➔ In the new version, we replaced “self-disparagement” by “self-derogation”. We thank the reviewer for this improvement.

Gender differences: I think the gender differences in the self-enhancement bias described in the paper are not entirely new and the authors should comment on them in the light of literature. For example, the authors can use the works on math-related self-enhancement of:

➔ We warmly thank the reviewer for the references. We use them in an additional paragraph devoted to gender differences in the discussion

Screening out participants: I would like to see some justification on using a broad RT filter based on 3 minutes. Why this value has been chosen? The literature on using response times in screening out careless respondents advocates for a different, more theory-based approach. The authors should present the distribution of response times and base their further moves on previous works, e.g.:

➔ We added this filter following the suggestion of a previous reviewer. The filter does not change the results and in the new version, we kept the results without filter in the main document. We added the filtered results in the supplementary materials.

Trust: Why the threshold of trust higher or equal to 7 was chosen? Would the results differ if other cut-off point have been picked up?

➔ We added the results for different levels of trusts in the main text. The higher the level of trust, the more significant the results are.

From very minor issues – the paper is in general well-written, but there are occasional typos and similar minor linguistic problems so I recommend proofreading before final submission.

➔ We tried to eliminate all these problems by several proof-readings. We made many minor changes throughout the whole text that are not marked.
