# Supplementary material for: A newly detected bias in self-evaluation
Source: PLoS One. 2024 Feb 8;19(2):e0296383. doi: 10.1371/journal.pone.0296383 (PMC10852250; doi:10.1371/journal.pone.0296383)
Supplement: Attachment — Submitted filename: answersToReviewers_R3.pdf [file pone.0296383.s015.pdf]

Dear Editor,

We thank you and the reviewers for your very valuable feedback on our paper.

Please find below the detailed explanations of the changes we made, answering point by point your requirements and the remarks or criticism from the reviewers. Our answers are in blue.

We think that the paper further improved and we hope that you and the reviewers will agree.

Kind regards.

Both reviewers still require a list of improvements. Some of the main points include:
- Justify the choice of responses provided to participants and clearly state the consequences of such choice for your findings

We think that the editor refers to the concern of reviewer #1 about some restrictions imposed on the answers of the participants. We expanded the second part of our explanation that was probably not clear enough.

- Justify/explain the sample size and undertake a power analysis

We performed a power analysis and added a paragraph in the paper reporting it.

- Theoretically justify all of your analysis and examine the findings accordingly

The editor probably refers to the part of the paper devoted to the effect of gender, self-esteem and scale on sensitivity and self-enhancement biases. In the introduction, we tried to clarify which part of this work could be related to the literature and which was more exploratory, because of the lack of references.

- Rethink your statistical approach in general, specifically consider using hierarchical modelling approach, if not applicable explain in details why and what are the limitations of not doing so

We added a hierarchical linear modelling analysis to the supplementary materials (different from the already proposed hierarchical linear models) and we explained why this method does not bring a very meaningful contribution in our context.

- Strengthen the Introduction theoretically (with works suggested by reviewers), but also the Discussion, including sections on future research and limitations

We expanded the introduction and the discussion, adding the additional references suggested by reviewer #2.

- Make a more substantial effort in providing figures that help in understanding the paper's findings

We replaced tables 4-7 by figures. We hope these are easier to understand.

Reviewer #1: This relatively thorough revision has improved the paper in several respects. I still have reservations which I will list briefly below, but the paper now seems to offer enough value to warrant publication. The empirical demonstration of a novel process leading to self-enhancement, evaluation-dependent sensitivity to feedback, is a solid contribution.

We thank the reviewer for this assessment.

The authors may or may not wish to address my remaining concerns.

1. The experiment forced participants to respond as theory specifies (making each self-assessment update fall between the previous self-assessment and the current feedback). The paper notes that in pilot testing, a majority of participants responded in this way without being restricted. However, the restriction still troubles me – it shapes the way participants think about the task, clearly pushing them in the direction of conforming to the theoretical prediction.

The reviewer does not react to the second argument that we recall here.

> self-evaluation is primarily a means to limit the noise in the results. Moreover, the way in which the sensitivity changes within the bounds, when the self-evaluation varies, is our main subject of investigation and it is not constrained.

The absence of reaction from the reviewer suggests that this second argument is not stated clearly enough. We modified it as follows:

> self-evaluation is primarily a means to limit the noise in the results. Moreover, the participants are free to choose any value within the bounds. The possible variation of this choice when the self-evaluation varies is thus constrained only in its limits, not in its direction. The main subject of our investigation is precisely the direction of this variation, which we expect to be decreasing. This direction of variation is not constrained by the experimental setting.

2. The primary hypotheses (line 69) are solid and well-motivated. In contrast, the secondary objective is to examine several potential moderator variables which are not introduced with any conceptual rationale. This is especially troubling for the evaluation scale, which has strong and unpredicted effects on the results. One previous study found a somewhat similar pattern, but no compelling theoretical interpretation can be offered in this paper (lines 490-518).

We tried to better motivate this part of the paper. We report the literature about the effect of gender and self-esteem in the introduction. We also explain that we tested two scales in order to check that the sensitivity bias was not an artefact coming from the scale. The results suggest that it is not. Further, this led us to test the effect of the scale on the self-enhancement. It seems that this effect is not reported in the literature and we explicitly recognise that these tests are thus exploratory.

3. I still do not think the data analysis strategy is optimal. First, participants who express low

“trust” in the experimental deception are irrelevant to the hypotheses and should not be included in the analysis. Second, the analyses in the main manuscript inappropriately use multiple observations ("triples") from each participant, which are statistically non-independent in violation of the assumptions of ordinary regression analysis. The analyses in SM use a two-step approach, running a regression for each participant and then predicting the coefficients of those analyses as outcome measures. This approach suffers from low power, because in the first step each analysis has very few observations (and so few coefficients are significant, line 18 of SM). More appropriate would be a hierarchical linear analysis, for example with the lmer function in R. Such an analysis does not use two separate steps but properly accounts for the non-independence of multiple observations from each participant, and gives results that describe the entire sample.

In our view, the two hierarchical linear models presented in the SM suggest that the data are independent enough (no individual or temporal clear trend) which authorises linear models mixing participants and time steps.

Moreover, we performed a standard hierarchical linear analysis that we added to the SM. The analysis shows that, for high levels of trust, the only significant variable is the self-evaluation. In any case, we think that this approach is not very meaningful for our aim and we explain it in the method section. Please find below the additional statement about it in the method section and the results of the hierarchical linear analysis for the two first time steps (in the SM). The results for the 3 first time steps of all time steps are similar and also available in the SM.

Adding other predictors (for instance age, gender, trust...) to the previous regressions (with change of self-evaluation as outcome and evaluation as predictor) does not provide information about the variation of the sensitivities when these additional predictors vary. Indeed, this goal can only be reached if the regression predicts the main parameter of the sensitivity, i.e. its slope, like in the hierarchical linear models proposed in the supplementary materials (sections 2.1 and 2.2). Nevertheless, multi-factor regressions of the change of self-evaluation are available in the supplementary materials (section 1).

1 Hierarchical multi-factor regressions

Tables 1, 2 and 3 show the results of two multi-factor regressions, adding predictors to the regression taking as outcome the change of self-evaluation ($\frac{|a^i_{t+1}-a^i_t|}{\delta^i_t}$) as outcome and the self-evaluation ($\frac{a^t_i}{100}$) as predictor that we use to approximate the sensitivity. In each table, the results of the top panel are derived from the data of the participants of all trust levels (trust in $[0, 10]$), the bottom panel from the data restricted high trust levels (trust in $[7, 10]$).

Table 1 shows the results for the two first time steps ($t \in (1:2)$), table 2 for the three first time steps ($t \in (1:3)$) and table 3 for the four time steps ($t \in (1:4)$).

The self-evaluation is the most significant factor in all cases. When considering only the participants of high trust, self-evaluation is the only significant factor. When considering all trust levels, trust and gender can be significant (weakly) and the anchor is also significant when $t \in (1:4)$.

Trust $\in [0, 10]$ and $t \in (1:2)$

	Model 1	Model 2	Model 3
Self-evaluation	-0.08^{**}	-0.09^{***}	-0.11^{**}
Trust		0.004^{**}	0.004^{**}
Gender		0.009	0.009
Age			-0.0004
Self-Esteem			0.01
Scale			0.0009
Anchor			0.0002
R-squared	0.004	0.007	0.008

Trust $\in [7, 10]$ and $t \in (1:2)$

	Model 1	Model 2	Model 3
Self-evaluation	-0.12^{**}	-0.12^{**}	-0.13^{*}
Trust		-0.0006	-0.0002
Gender		-0.009	-0.008
Age			-0.0004
Self-Esteem			0.007
Scale			0.01
Anchor			-0.00001
R-squared	0.008	0.009	0.01

$***: p < 0.001$, $**: p < 0.01$, $*: p < 0.05$, $.: p < 0.1$

Table 1. Multi-factor regressions with change of self-evaluation ($\frac{|a^i_{t+1}-a^i_t|}{\delta^i_t}$) as outcome and three different sets of predictors. Top panel: Trust $\in [0, 10]$. Bottom panel: Trust $\in [7, 10]$. For both panels, $t \in (1:2)$.

Following the reviewer's recommendation, we kept only the results for the participants reporting a high trust in the section about the effect of scale, gender and self-esteem on the biases.

As stated above, however, despite these conceerns I think the paper offers meaningful value to the field.

We thank the reviewer for this assessment.

Reviewer #2: I thank the authors for addressing my comments.

I think that the present version of the article is markedly better than the previous one. Well done!

We thank the reviewer for this encouraging tone.

However, I still think that some parts of the manuscript should be improved before publication. I present my concerns below:

1. Please provide power analysis to justify that the sample sizes enable achieving required statistical power in your analyses.

We added the following paragraph at the end of the "Result treatment" section:

Power analysis

The power analysis concerns the linear regressions that approximate the sensitivity to feedbacks in different data sets. Using the G*Power software for linear regressions, with one tail, seeking a small effect (0.1 recommended by the software) and a power $(1 - \alpha)$ of 0.95, we get a recommended sample size of 1073.

We need to perform linear regressions for both positive and negative feedbacks and at least when considering each scale independently. This leads to a recommended sample size of 4*1073 = 4302.

We decided to collect a sample from 1500 participants, generating, with 4 triples each, a sample of size 4*1500 = 6000. The surplus of around 30% accounts for the unavoidable unreliable data (participants not believing in the feedbacks or with self-evaluations that make the feedbacks leave the interval [1, 100]).

2. The introduction still needs improving. I think the authors should clearly introduce topics such as agency and communion (with relation to self-enhancement) or different types of self-enhancement (impression management, self-deceptive denial, self-deceptive enhancement). Please elaborate more on that in the introduction, e.g., by using many excellent works of Delroy Paulhus (types of self-enhancement) or Bogdan Wojciszke (agency and communion). Please also refer to this recent article that seems relevant and also comments on inter-gender differences:

We thank the reviewer for this new reference. We added the following paragraph about self-enhancement.

The research distinguishes two main types of self-enhancement:

- Self-enhancement by impression management is self-promotion and positive self-presentation in a social context. It can be related to the need for approval [10] or the ability to monitor demands from the social environment [11].
- Self-enhancement by self-deception and narcissism designates unrealistic positive views of the self, illusions of control and unrealistic optimism. This includes self-deceptive overly positive self-perceptions [12, 13] and denial of having negative traits or socially deviant tendencies [14].

Moreover, the self-deceptive enhancement is related to two fundamental ways of obtaining social approval and reward: Agency which emphasises competence, independence and openness (get ahead) and communion which emphasises respect of

duty, interdependence, agreeableness (get along) [13, 15, 16]. This paper relates to self-deceptive enhancement in its various dimensions.

3. Please also include more information about the relations between self-esteem, gender and perceived level of expertise with self-enhancement in the introduction. An excellent paper by Stav Atir can help you to achieve this:

We added the following paragraph, presenting our secondary objectives in more details, in which we introduced some literature about self-enhancement and self-esteem or gender.

Our secondary objective is to observe how both sensitivity and self-enhancement biases are moderated by variables such as evaluation scale, gender and self-esteem. Of course, there is no existing literature about the effect of these variables on the sensitivity bias, as this bias has not been detected yet and is completely different from the already studied biases. Hence this part of the paper can be seen as exploratory.

Surprisingly, we did not find any previous theoretical or experimental work about the effect of the evaluation scale on self-enhancement and our results about this effect can also be seen as exploratory. By contrast, there is a very abundant literature about the effects of gender and self-esteem on self-enhancement. Obviously, it is out of the scope of this paper to provide an exhaustive review of this literature and we limit our analysis to a restricted set of selected references that appear particularly relevant in the context of our experiment.

From this analysis, that we report in more details in the discussion, we conclude that:

- The literature points to an increase of self-enhancement with self-esteem. The main rationale behind this effect is that people with high self-esteem tend to be more motivated to protect or increase their self-esteem [28, 29] or high self-esteem tends to increase the self-deceptive mechanisms of self-enhancement [30].
- In the case of our experiment, the literature predicts that men will self-enhance more than women. Indeed, first, in our experiment, the task is related to a rather masculine subject and men tend to self-enhance more than women with respect to such subjects [31, 32]. Second, the evaluation is based on individual performance,

closely related to the agency domain, in which men tend to self-enhance more than women [13,33]. Finally the performance is not publicly disclosed, hence the self-enhancement is rather self-deceptive and men are more easily subject to this self-deception than women who engage more easily in impression management [34].

With respect to our secondary objective, our main finding is that the average sensitivity bias remains relatively stable when scale, gender and self-esteem vary (in the group of participants who believed in the feedbacks). This stability of the sensitivity bias is remarkable because the closely related self-enhancement bias varies very significantly, primarily with the scale.

Indeed, when the scale decreases with the evaluation, we find a significant self-enhancement bias as expected, but when the scale increases with the evaluation, we systematically find a significant self-derogation bias, like in the experiment of [25] (who used an increasing scale). This effect, which, as far as we know, has not been reported in the literature, questions the explanation of the self-derogation by the motive to learn proposed in [25].

Moreover, in line with our analysis of the literature, the group of participants with high self-esteem and men tend to show a higher self-enhancement or a significantly weaker self-derogation.

4. Please, please think hard on how can you improve the presentation of your findings in a graphical way. For now, it is hard to follow the information flow presented in Tables 4-7. I would also like to point your attention to the fact that they do not conform to any widely accepted style. I would suggest to rethink their "look" and redraw them in the APA style. This also relates to the * for p-levels. Still, I do not know to which number of stars is related to which p-level.

We changed the format of the tables. Please see below. We repeated the meaning of the significance codes below both tables (we previously put it only below the first table and it seems that the reviewer missed it). We also put all the tables of the Supplementary materials in this format.

Slope of sensitivity to feedback on several time steps

Trust	$t \in (1:2)$		$t \in (1:3)$		$t \in (1:4)$	
	N	c	N	c	N	c
$[0, 10]$	2736	−0.08**	4104	−0.09***	5472	−0.07***
$[0, 6]$	1656	−0.07*	2484	−0.07*	3312	−0.04 .
$[7, 10]$	1080	−0.12**	1620	−0.13***	2160	−0.11***
$[8, 10]$	834	−0.16***	1251	−0.15***	1668	−0.13***
$[9, 10]$	562	−0.18**	843	−0.18***	1124	−0.16***

*** : $p_v < 0.001$, ** : $p_v < 0.01$, * : $p_v < 0.05$, . : $p_v < 0.1$

Table 2. Slope of sensitivity to feedback on several time steps. The slope is computed for different intervals of trust. N is the number of triples $(a_t^i, f_t^i, a_{t+1}^i)$ in the considered data set.

Trust	N	$c(1)$	$c(2)$	$c(3)$	$c(4)$
[0, 10]	1368	−0.09*	−0.07 .	−0.09*	−0.01
[0, 6]	828	−0.08 .	−0.06	−0.06	0.02
[7, 10]	540	−0.13*	−0.12*	−0.13*	−0.07
[8, 10]	417	−0.14*	−0.18**	−0.13 .	−0.1
[9, 10]	281	−0.2*	−0.16 .	−0.19*	−0.09

Slope $c(t)$ of sensitivity to feedback at each time step

** : $p_v < 0.01$, * : $p_v < 0.05$, . : $p_v < 0.1$

Table 3. Slope $c(t)$ of sensitivity to feedback at each time step. The slope is computed on data from participants reporting different trust values (first column). N is the number of triples $(a_t^i, f_t^i, a_{t+1}^i)$ in the considered data sets (it does not vary with t).

Moreover, we replaced tables 4-7 by figures. We simplified the presentation of these results by focusing only on the participants a high level of trust (in [7,10]). We reproduce these figures below (same numbering as the tables previously, except that we inverted 6 and 7)

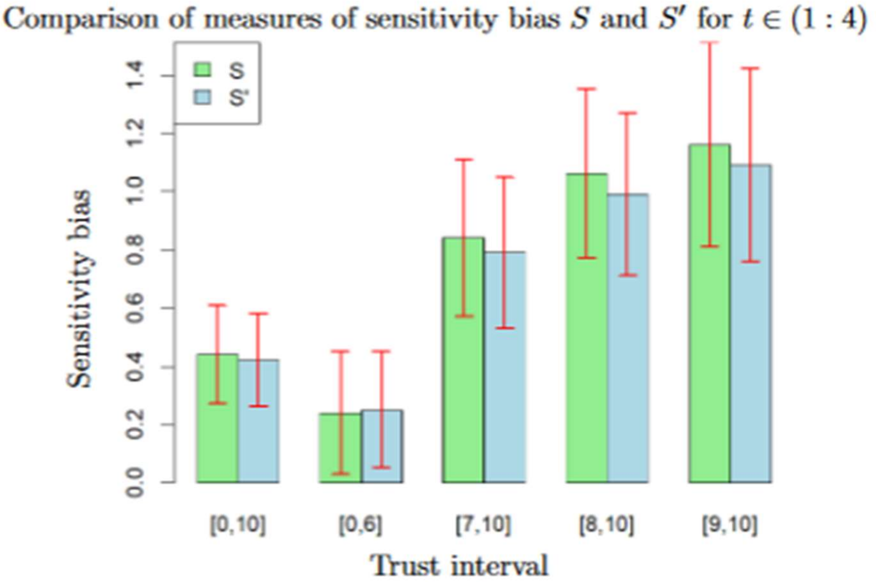

Figure 4. Comparison of measures S and S' of the bias from sensitivity to feedbacks for $t \in (1:4)$. Both measures are computed on data from participants reporting trust in an interval. The value and the error bars are the average and standard deviation computed on 200 bootstrap samples.

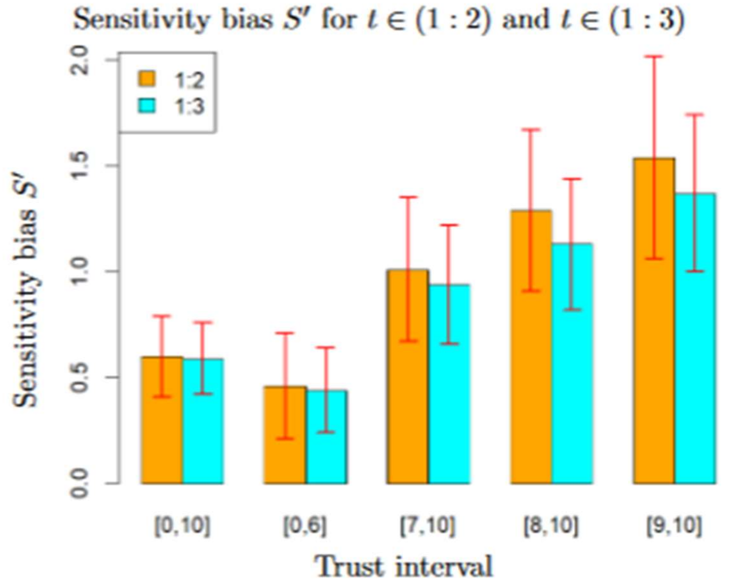

Figure 5. Measure S' of the bias from sensitivity to feedbacks for $t \in (1:2)$ and $t \in (1:3)$. The values and the error bars are the mean and standard deviation computed on 200 bootstrap samples.

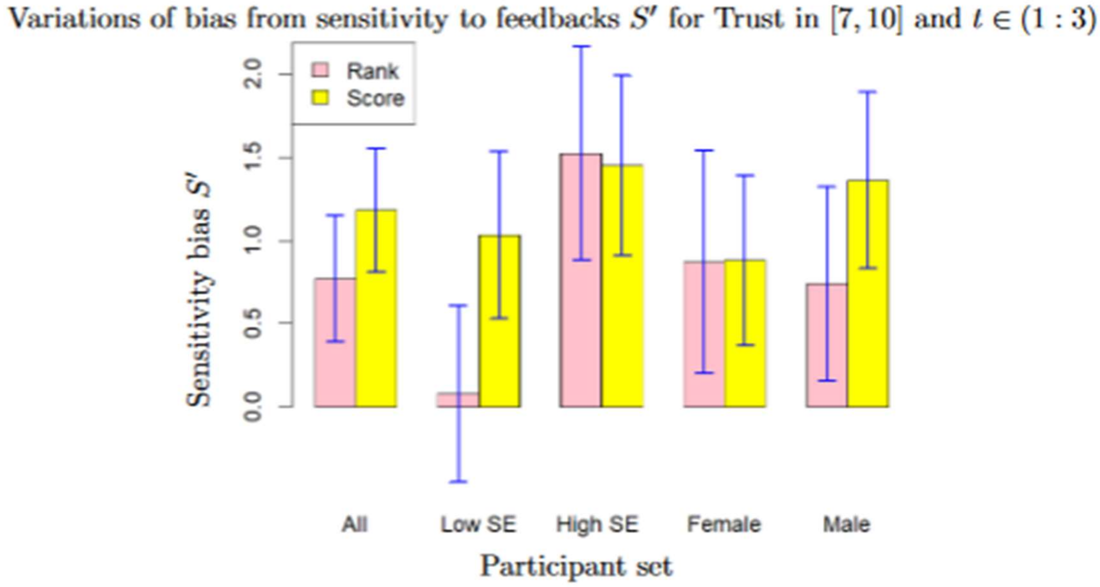

Figure 6. Sensitivity bias E for different values of scale, gender and self-esteem (SE), for trust in $[7, 10]$ and $t \in (1:3)$. The bars and the error bars are respectively the mean and standard deviation computed on 200 bootstrap samples.

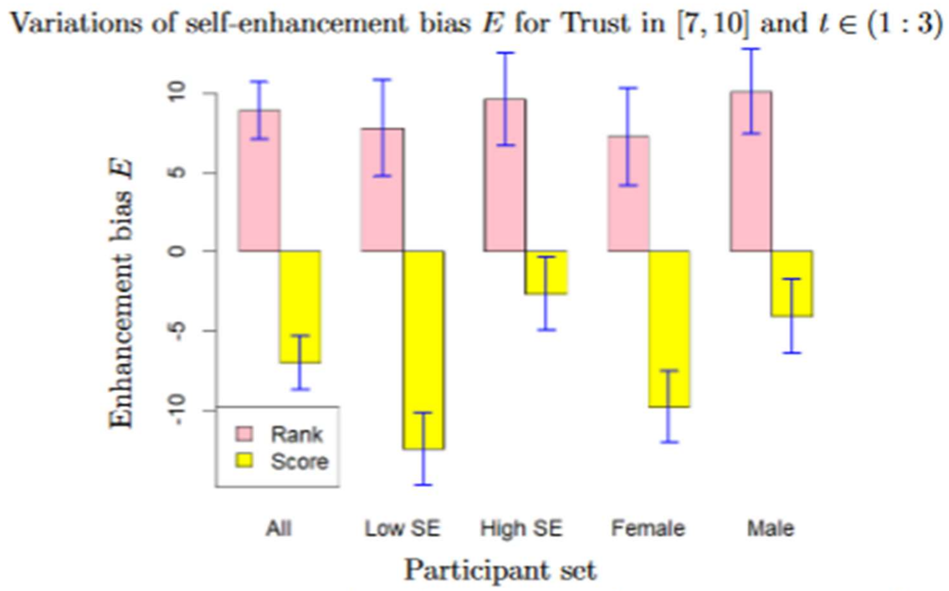

Figure 7. Enhancement bias E for different values of scale, gender and self-esteem (SE), for trust in [7, 10] and $t \in (1:3)$. The bars and the error bars are respectively the mean and standard deviation computed on 200 bootstrap samples.

5. I was also expecting some results about the role of gender and self-esteem play in feedback sensitivity and self-enhancement presented more in a fashion of regresssion results. Could you possibly present it in this way to increase understanding of the analyses and results?

We added this type of regressions in the supplementary materials. We explained why they are not very appropriate in our approach in the method section. Please see below this explanation and the results of the regressions.

Adding other predictors (for instance age, gender, trust...) to the previous regressions (with change of self-evaluation as outcome and evaluation as predictor) does not provide information about the variation of the sensitivities when these additional predictors vary. Indeed, this goal can only be reached if the regression predicts the main parameter of the sensitivity, i.e. its slope, like in the hierarchical linear models proposed in the supplementary materials (sections 2.1 and 2.2). Nevertheless, multi-factor regressions of the change of self-evaluation are available in the supplementary materials (section 1).

1 Hierarchical multi-factor regressions

Tables 1, 2 and 3 show the results of two multi-factor regressions, adding predictors to the regression taking as outcome the change of self-evaluation ($\frac{|a^i_{t+1} - a^i_t|}{\delta^i_t}$) as outcome and the self-evaluation ($\frac{a^i_t}{100}$) as predictor that we use to approximate the sensitivity. In each table, the results of the top panel are derived from the data of the participants of all trust levels (trust in $[0, 10]$), the bottom panel from the data restricted high trust levels (trust in $[7, 10]$).

Table 1 shows the results for the two first time steps ($t \in (1:2)$), table 2 for the three first time steps ($t \in (1:3)$) and table 3 for the four time steps ($t \in (1:4)$).

The self-evaluation is the most significant factor in all cases. When considering only the participants of high trust, self-evaluation is the only significant factor. When considering all trust levels, trust and gender can be significant (weakly) and the anchor is also significant when $t \in (1:4)$.

Trust $\in [0, 10]$ and $t \in (1:2)$

	Model 1	Model 2	Model 3
Self-evaluation	-0.08^{**}	-0.09^{***}	-0.11^{**}
Trust		0.004^{**}	0.004^{**}
Gender		0.009	0.009
Age			-0.0004
Self-Esteem			0.01
Scale			0.0009
Anchor			0.0002
R-squared	0.004	0.007	0.008

Trust $\in [7, 10]$ and $t \in (1:2)$

	Model 1	Model 2	Model 3
Self-evaluation	-0.12^{**}	-0.12^{**}	-0.13^{*}
Trust		-0.0006	-0.0002
Gender		-0.009	-0.008
Age			-0.0004
Self-Esteem			0.007
Scale			0.01
Anchor			-0.00001
R-squared	0.008	0.009	0.01

$*** : p < 0.001$, $** : p < 0.01$, $* : p < 0.05$, $. : p < 0.1$

Table 1. Multi-factor regressions with change of self-evaluation ($\frac{|a^i_{t+1} - a^i_t|}{\delta^i_t}$) as outcome and three different sets of predictors. Top panel: Trust $\in [0, 10]$. Bottom panel: Trust $\in [7, 10]$. For both panels, $t \in (1:2)$.

6. In lines 75-76 and 187 you use the term "modulated". I think you should use "moderated" or "related to", depending on what is your analytic stance towards those relations. "Modulated" is a conversational term and should not be used here (I know it is used widely, but I think we should avoid it).

Done

7. Please tone down the language in lines 58-63. These are only hypotheses/suggestions.

We changed the text as follows:

[illegible]

As shown in this paper, a positive bias in self-evaluation appears as a direct mathematical consequence of the decreasing sensitivity to the feedback. Therefore, if its existence is confirmed, this bias would be disconnected to any motive for ego enhancement, preservation or improving. It would simply be the mechanical side-effect of a general property of the sensitivity to the feedback. As far as we know, the literature does not mention this bias yet. We call it bias from decreasing sensitivity to [illegible]

8. Regarding lines 100-107 - can you provide any evidence from studies with human participants?

These models are theoretical and the papers do not cite any experimental evidence to ground the hypotheses.

9. Short question about Table 1 - why does it contain only four combinations? There are more combinations of balanced six feedbacks...

The table shows the six possible combinations of balanced four feedbacks.

10. It is outstanding that you have found such a valuable and relevant references as number 19 (Muller-Pinzler et al., 2019)! I think you should capitalise more on that and present this important study more in the introduction. Please, do not assume that the readership knows this paper. It should be sufficiently presented and commented on in the relevant sections of the paper.

We expanded the description of the experiment of (Muller-Pinzler et al., 2019) (ref [25] in the second revision).

If self-enhancement seems to be a generally dominant motive, in some contexts the motives of self-assessment, self-confirmation or self-improving may prevail [9,24]. In this respect, the experiment reported in [25] is an important example for this paper because our own experiment shares several features of its design. Surprisingly, the results of [25] show that the participants give more influence to negative feedbacks than to positive ones, hence they show a negative bias. This experiment repeats sequences where the participants perform a task then receive a feedback about their performance at the task and try to improve their performance at the next sequence. The feedback is actually defined by the experimenters and completely disconnected from the performance of the participants. The authors argue that because the participants are learning a task and try to improve their performance, negative feedbacks are more informative and thus are given more weight, which induces a negative bias. The authors suggest that it should generally be the case in a repetitive learning context.

This led us to describe a bit more our own experiment and its results in the introduction, as follows:

In this paper, our primary objective is to bring experimental evidence supporting two main hypotheses:

1. People show an average decreasing sensitivity to feedbacks, when their self-evaluation increases;
2. This induces a specific positive bias in self-evaluation that is added to self-enhancement (or self-derogation) bias.

We report the results of an experiment (N = 1509) designed with this aim. The participants in this experiment perform a task once and then evaluate their performance at the task several times in reaction to evaluations (feedbacks) given by the experimenters. We use two scales for the evaluation, because it is important to check that the potential sensitivity bias is not an artefact coming from the scale. The first scale increases with the performance like in [25], the second scale decreases with the performance. From data coupling self-evaluation and change of self-evaluation in reaction to a feedback, we compute a linear regression approximating the average sensitivity to feedbacks as a function of self-evaluation in different sets of participants. The slope of this linear regression is significantly negative especially when computed from the participants who believed that the feedbacks were real, showing that the sensitivity to feedbacks is decreasing. Moreover, we measure a significant average positive bias from this decreasing sensitivity to feedbacks, additional to the self-enhancement (or self-derogation) bias, especially in the set of participants who believed that the feedbacks were real. These results provide evidence supporting our hypotheses.

11. Please expand the discussion on self-esteem and self-enhancement on topics such as sensitivity to feedback, narcissism, fragile self-esteem and rely to relevant literature.

We completed the text of the first revision as follows:

Effect of self-esteem

Self-enhancement and self-esteem are deeply related as the self-enhancement motive is to preserve or increase self-esteem. Yet, the literature shows contradictory views about the influence of self-esteem on self-enhancement [35]. The self-enhancement theory for instance assumes that individuals with a low self-esteem have a stronger motivation for self-enhancement [36]. Other theories suggest that, on the contrary, individuals with a high self-esteem are more motivated to protect their positive self-view [37, 38] or to confirm it [25]. The experiments reported in [39] corroborate the latter theories, as they suggest that the status of expert provides enough overconfidence to claim impossible knowledge. The experiment reported in [25] corroborates them as well, as participants with a lower self-esteem tend to show a more significant negative bias. Similarly, in our results, sets of participants with a lower self-esteem tend to show a greater self-derogation (for the evaluation by score) and a lower self-enhancement (for the evaluation by rank).

Effect of gender

Previous research established gender differences in self-enhancement. First, men tend to engage more, comparatively to women, in self-deceptive enhancement and women more in impression management [39]. Our experiment does not involve much impression management, as the participants are told that they interact, via the computer, with a program that computes their rank or score in several predefined groups. This can explain the lower self-enhancement of women measured in our experiment.

Also, the gender difference may depend on the context. In general, men reveal significantly higher self-enhancement with respect to masculine subjects than women do, whereas the self-enhancement of men and women in relation to feminine subjects are [illegible]

We are conscious that the reviewer is probably expecting more expansion of the text. However, like for the introduction, we are reluctant to expand the paper too much in the direction of self-enhancement, as it is not our main topic and half the participants in the experiment show self-derogation, not self-enhancement. We hope that the reviewer can understand our point of view.

12. The paper lacks important sections such as "Limitations" and "Future directions". I would ask authors to amend for that.

Following the reviewer's suggestion, we added the following subsection:

Limitations and future challenges.

A major limitation of the experiment is the small size of data obtained from each participant. Indeed, the sensitivity to feedback may vary significantly with the participants and computing average sensitivity functions in sets of participants hides this variety. Ideally, the experiment should collect a much larger number of triples (self-evaluation, feedback, change in self-evaluation) from each participant in order to derive significant individual models of sensitivity to feedbacks.

A second limitation worth mentioning relates to the detection of the sensitivity bias itself. Indeed, its detection in a very specific experiment says nothing about its potential role in daily life. Actually, our measures suggest that the sensitivity bias

might generally be too dominated by self-enhancement or self-derogation biases to show an independent effect. Moreover, the agent based model simulations suggest that the sensitivity bias has a significant effect only on the long term. In this case again, long series of data could provide more information on the potential effect of this bias.

The setting of our experiment is clearly inadequate for collecting long series of individual data as the attention and motivation of participants already drop at the fourth time step. Designing a completely different experiment, that would provide long term individual data, is a serious challenge. Large scale long lasting game experiment on the internet, like for instance the one reported in [46], could offer new means to address this issue.

Finally, it seems noticeable that our main result, the existence of a bias from sensitivity to feedbacks, originates in theoretical agent simulations. This bias was indeed identified because its effects were easily observed in long lasting simulations, involving millions of virtual interactions. We could then detect its much smaller effect on short simulations, that we had initially overlooked. Similarly, it seems almost impossible to observe this bias in real life without looking for it with specific computations on data from a specific experiment. This is a case, common in physics but not so much in social sciences, of an initially purely theoretical concept whose existence is confirmed experimentally.

A second case of theoretical bias could be experimentally confirmed in the near future. Indeed, the theoretical work on the agent model identifies a second bias from the decreasing sensitivity to feedbacks, a negative bias on the evaluation of others [26, 27]. This bias has also not been observed yet and designing a new experiment to detect it is another serious challenge. The experiment reported in this paper seems an interesting starting point to take up this challenge.

I see that the paper is approaching the right direction and would like to encourage the authors for some more effort before the publication. Fingers crossed!

We thank the reviewer for this encouraging comment.
