# Supplementary material for: A newly detected bias in self-evaluation
Source: PLoS One. 2024 Feb 8;19(2):e0296383. doi: 10.1371/journal.pone.0296383 (PMC10852250; doi:10.1371/journal.pone.0296383)
Supplement: Attachment — Submitted filename: answerToReviewersR4.pdf [file pone.0296383.s016.pdf]

Dear Editor,

We thank you and the reviewers for your valuable feedback on the second version of the paper.

In this third version, we computed linear mixed effect models and we mentioned the restriction in the values of self-evaluation that the participants express as a limit of the experiment as you requested, following the comments of reviewer#1.

Moreover, we tried our best to answer the different requests from reviewer#2.

In the following, we reproduced the remarks of the reviewers in black and our answers are in red. We also reproduce the parts of the manuscript with changes (appearing in blue).

Respectfully,

Guillaume Deffuant

Reviewer #1: This second revision is very similar in substance to the previous version. The theoretical point that differential sensitivity to feedback is a novel mechanism that can contribute to self-enhancement is sound. But I still have two significant concerns about the experimental demonstration reported in this paper.

First, as noted before: "The experiment forced participants to respond as theory specifies (making each self-assessment update fall between the previous self-assessment and the current feedback). The paper notes that in pilot testing, a majority of participants responded in this way without being restricted. However, the restriction still troubles me – it shapes the way participants think about the task, clearly pushing them in the direction of confirming the theoretical prediction." The authors' response emphasizes that this restriction was intended to reduce noise in the observations, and that participants remained free to choose any self-evaluation between the given limits. But that response misses my point that the restriction imposes on participants a specific way of thinking about the task. By forcing them to think of it as updating their previous response by moving some proportion of the way toward the current feedback, the researchers are in effect forcing them to use the theoretically assumed process. There are potentially other ways participants might approach the task if this restriction was not artificially imposed, for example deciding that the feedback jumps around too much to be useful or believable and therefore trying to disregard it. The authors' response completely ignores this concern, that the restriction shapes the way participants think about the task.

Our main point is that the restriction does not constrain the change of sensitivity when the self-evaluation varies, which the main topic of our work. Therefore, if the sensitivity decreases when the self-evaluation increases with the restriction, the same phenomenon should also take place without the restriction.

Nevertheless, we acknowledge that it would be important to confirm this assumption by replicating the experiment without the restriction. We therefore added the following point in the limitations section:

derive significant individual models of sensitivity to feedbacks. Moreover, in the experiment, we constrain the self-evaluations to be between the previous self-evaluation and the feedback. We underlined that this constraint on the expression of self-evaluations does not constrain the change of sensitivity when the self-evaluation varies, therefore, as we find a decrease of this sensitivity when self-evaluation increases with the constraint, we should also find it without the constraint. Nevertheless, it would be important to check this assumption by replicating the experiment without the constraint. This would certainly require a larger sample to cope with more noisy data.

Please note that the restriction does not prevent the participants to disregard the feedback and we observe instances of this disregard in the data.

Second, I remain concerned that the main analyses use ordinary linear regression on a set of "triples," which include multiple observations from each participant. Those are obviously non-independent, violating a statistical assumption of the regression model. I recommended using a hierarchical regression (also termed multilevel or mixed model), in which participants are explicitly treated as a random effect, to correctly account for this non-independence.

The authors respond in two ways. One part of the authors' response is to present regression analyses that include additional predictors (age, gender, trust, etc.). These are entirely irrelevant to the criticism I was making.

Second, they report "hierarchical" models in SM (2.1 and 2.2) that they argue show any non-independence can be ignored. These models are analyzed in two separate steps (between and within participants) rather than as a standard hierarchical model, for example using lmer* in R. I do not understand how or why the authors claim that the results from these models rule out non-independence. More important, experts recommend the use of hierarchical models in all cases when the data has a multilevel structure (here, observations nested within participants) regardless of the statistical level of non-independence. For example, Nezlek (2008)* writes "analysts should use multilevel modeling when they have a multilevel data structure – pure and simple. When I am asked for advice regarding whether or not multilevel modeling is appropriate, my first question concerns the nature of the data structure. If there is a meaningful nested hierarchy to the data, my advice is to use multilevel modeling, irrespective of distracting arguments about ICCs and so forth." And "researchers sometimes use a low or zero ICC to justify a decision not to use multilevel modeling – on the grounds that because there is no (or very little) between-group variance in the dependent measure, the grouped (or nested) structure of the data can be ignored. This is a dangerous assumption that is not justifiable. Frequently (or almost invariably), researchers are interested in relationships between measures. The fact that there is little or no between-group variance in a measure does not mean that the relationship between this measure and another measure is the same across all groups, something that is assumed if one conducts and analysis that ignores the grouped structure of the data. By extension, even if there is no between-group variance for all of the measures of interest, it cannot be assumed that relationships between or among these measures do not vary across groups."

To be absolutely clear, the model I view as appropriate, expressed as an lmer* formula, is something like:
Dependent.variable ~ 1 + independent.variables + (1 + independent.variables | Participant)
where the term in parentheses indicates random slopes and intercepts per participant.

We warmly thank the reviewer for taking the time to write this detailed explanation, providing us with all the information for applying the adequate statistical method.

Using this method led us to the following changes in this third version of the paper.

First, we added the following paragraph in the section "Result treatment", in subsection "Linear approximations of the sensitivity to feedback functions":

When the sets include 3 or 4 time steps, we also computed linear mixed effect models, using the R package **lme4** [39] for the approximation of the sensitivity to feedbacks (positive and negative together), because the 3 or 4 self-evaluations of a single participant are not independent [40]. In the other cases, sets including less than 3 time steps, or including only positive or only negative feedbacks, the linear mixed effect model is not applicable and we use only standard linear regressions (see more details in the supplementary materials, section 1). ~~Adding other predictors (for instance age,~~

Second, in the supplementary material, we added the following section including more details about linear mixed effect models.

1 Linear mixed effect models

Linear mixed effect models are relevant when the data are structured in different levels [1]. In particular, in our data, each participant generates 4 triples including self-evaluation, feedback and self evaluation change. The independence hypothesis between these values is not verified as they come from a single participant. The linear mixed effect method defines a linear approximation which takes the structure into account. It outputs fixed effects (or coefficients) that are the coefficients of a linear model, taking the structure into account, and random effects corresponding to a variance of these coefficients when the structure varies. We focus our analysis on the fixed effects which we expect to provide a sound linear approximation of the sensitivity to feedbacks. In practice, we use the `lmer` function from the `lme4` R package [2]. The typical function call is:

```
model = lmer(|deltaEval| ~ eval + (eval | participant), data),
```

where `deltaEval` is the change of self-evaluation and `eval` the self-evaluation. This method is applicable only if there are more than two values of triples (self-evaluation, feedback and self evaluation change), otherwise, there is no meaningful second level. Therefore, we can use the `lmer` function only when including both positive and negative feedbacks and at least 3 time steps for each participant. This implies that the function cannot be used to compute the different measures of biases, as these measures require separating positive from negative feedbacks and there are only two time steps of each for each participant.

Third, in the result section, we modified the first subsubsection "The sensitivity to feedback decreases when self-evaluation increases". In table 2, we added the slopes computed with the lmer R function, for the three first time steps and all four time steps, for different levels of trust. Table 2 becomes:

Slope of sensitivity to feedback on several time steps

Trust	$t \in (1:2)$		$t \in (1:3)$			$t \in (1:4)$		
	N	c	N	c	c(lmer)	N	c	c(lmer)
[0, 10]	2736	-0.08^{**}	4104	-0.09^{***}	-0.08	5472	-0.07^{***}	-0.06
[0, 6]	1656	-0.07^{*}	2484	-0.07^{*}	-0.07	3312	-0.04 .	-0.04
[7, 10]	1080	-0.12^{**}	1620	-0.13^{***}	-0.12	2160	-0.11^{***}	-0.1
[8, 10]	834	-0.16^{***}	1251	-0.15^{***}	-0.14	1668	-0.13^{***}	-0.12
[9, 10]	562	-0.18^{**}	843	-0.18^{***}	-0.17	1124	-0.16^{***}	-0.14

$*** : p < 0.001$, $** : p < 0.01$, $* : p < 0.05$, $. : p < 0.1$

Table 2. Slope of sensitivity to feedback on several time steps. The slope is computed for different intervals of trust. N is the number of triples $(a_t^i, f_t^i, a_{t+1}^i)$ in the considered data set. c is the slope computed with a standard linear regression and c(lmer) designates the slope computed with a linear mixed effect model (p-value not provided).

We made some changes of the text related to these additions. The beginning of this subsection becomes (additions in blue):

The sensitivity to feedback decreases when self-evaluation increases

Our first hypothesis is that the sensitivity to feedbacks decreases when self-evaluation increases. This sensitivity is evaluated by the linear regression taking change of self-evaluation as outcome variable and self-evaluation as predictor variable (equation 29), that we recall here for convenience:

$$\frac{|a_{t+1}^i - a_t^i|}{\delta_t^i} \approx c \frac{a_t^i}{100} + b. \qquad (37)$$

We also evaluate the slope c using a linear mixed effect model, which takes the non-independence of the self-evaluations from the same participants into account (see supplementary material section 1 for details).

Table 2 shows the value of the slope of the sensitivity c derived with a standard linear regression from different data sets mixing several time steps ($(1:k) = \{1, .., k\}$) and participants of different values of trust in the feedback (on the first line of the table all the possible values of trust are considered). For the three first time steps or all the four time steps ($t \in (1:3)$ or $t \in (1:4)$), the table also shows the slope computed with

the linear mixed effect model (c (lmer)). Note that the method does not provide a p-value associated with the slope. The table also shows the size N of each data set.

In all cases, the value of the slope c is significantly negative, with an increasing amplitude when trust in the feedback increases. The slopes computed with the linear mixed effect model are very close to the slopes obtained by standard linear regression (only slightly less negative). Therefore, the non-independence of the self-evaluations for each participant has a weak effect.

Overall these results confirm our main hypothesis that sensitivity to feedback decreases when self-evaluation increases. The confirmation is particularly clear in sets of participants reporting a high trust, but the hypothesis is also valid in those reporting a low trust. Figure 3 represents two examples of sensitivity to feedbacks computed on sets of participants with high trust.

Fourth, we removed the previous section about hierarchical models from the supplementary material, as it is no longer relevant. We also removed all references to this section in the main text.

Fifth, we also removed the section about linear models including other predictors than the self-evaluation from the supplementary material, as we also think that it is not relevant. We removed all references to this section in the main text.

Reviewer #2: I think that the manuscript is improved but it is still written in a unnecessarily complicated way, even convoluted sometimes. The literature review is still shallow at times, although the changes introduced in the review rounds have increased the quality and comprehensibility of the paper.

I feel that I cannot provide more suggestions for the authors and it is up to the editors to decide on further fate of the manuscript. I just have few remaining suggestions/questions (the number is the number of line in the corrected manuscript):

a) 28-29 "in its various dimensions"
I think the paper relates to self-enhancement in the sense of self-exaggeration?

We removed “in its various dimensions”

b) 109-112
Is it really completely different from other biases?
Please tone down the language here as the originality of the effects is not as high as you claim.
Take a look at feedback sensitivity research available, e.g. this article by Filip Gęsiarz:
Gesiarz, F., Cahill, D., & Sharot, T. (2019). Evidence accumulation is biased by motivation: A computational account. PLOS Computational Biology, 15(6), e1007089.

This paper shows mainly that positive feedbacks are given more importance by the participants, which is the standard mechanism of the enhancement bias in our view. There is no mention of a decreasing sensitivity to feedbacks when self-evaluation (which is not

measured) increases, as a mechanism for a different bias. Therefore, we do not see any relation between this paper and the bias that we claim to have detected.

This topic was also researched by Stav Atir or Jennifer S. Beer, for example.

In the paper of Atir and Dunning (When Knowledge Knows No Bounds: Self-Perceived Expertise Predicts Claims of Impossible Knowledge. Psychological Science. 2015;26(8):1295–1303), the overconfidence is almost certainly not due to the bias from decreasing sensitivity because it would then be moderate or small (second order of the feedback), which is not the case. We can imagine that the status of expert leads to a strong decrease of the sensitivity to the negative feedbacks, which increases the standard self-enhancement bias. There is probably also a smaller decrease of sensitivity to positive feedbacks. However, the effect of the overall decrease of the sensitivity to feedbacks, generating a positive bias which comes in addition to the self-enhancement bias, is completely absent from the paper. Again, we see no link between this paper and the bias that we claim to have detected.

In the paper of J. Beer (Exaggerated Positivity in Self-Evaluation: A Social Neuroscience Approach to Reconciling the Role of Self-esteem Protection and Cognitive Bias. Social and Personality Psychology Compass. 2014;8(10):583–594), the main point is showing that exaggerated positivity protecting self-esteem and exaggerated positivity not protecting self-esteem activate different neuronal circuits. We do not see any reference to the decreasing sensitivity to feedbacks as a possible mechanism generating a specific bias, different from the usual self-enhancement bias.

We did not find any publication mentioning the decreasing of the sensitivity to feedback as causing a specific positive bias in self-evaluation. Nevertheless, it is impossible to be completely sure that we did not miss any. Therefore, we toned down our statement as follows:

> Our secondary objective is to observe how both sensitivity and self-enhancement biases are moderated by variables such as evaluation scale, gender and self-esteem. Of course, ~~there is no existing literature~~ we did not find any publication about the effect of these variables on the sensitivity bias, since, as far as we know, this bias has not been detected yet and is completely different from the already studied biases. Hence this part of the paper can be seen as exploratory.

c) 303
Table 1 says "six series of 4 feedbacks" but I rather see four series of six different feedbacks?

We realise that the reviewer rather sees four series of six different feedbacks probably because of the vertical lines in the table. We removed these vertical lines and put the table into an APA like format (see below). We hope that this solves the problem.

f_1	f_2	f_3	f_4
+	+	-	-
+	-	+	-
+	-	-	+
-	+	+	-
-	+	-	+
-	-	+	+

Table 1. The six series of the 4 feedbacks f_1, f_2, f_3 and f_4 (two positive, two negative).

d) 476
you do not have sample size of 6000, you just have 1500 that you use in 4, if I am right, different analyses, n'est-ce pas?

Pas vraiment, avec tout notre respect.
We compute the linear approximation of the sensitivity to feedbacks in sets mixing all the time steps (1:4) (so all the 5472 triples all together when taking all trust levels), the three first time steps (1;3) (4004 triples together), the two first (1:2) (2736 triples) and each time step considered in isolation (1368 triples) (and in all cases also for different trust values which lowers the number of triples).

For the analysis of the biases (from self-enhancement and from sensitivity), we mixed the three first time steps (1:3), because there is evidence that the data from the last time step are less reliable. However, the same analyses for time steps 1:2 and 1:4 are available in the supplementary material.

e) 553
I think that treating continuous variables as dichotomous is very questionable, I advice to redo the analyses presented in Figure 6 with trust and self-esteem treated as continuous predictors

Unfortunately, we cannot do that because computing the sensitivity bias (or the self-enhancement bias) requires performing two linear regressions (one on the positive, the other on the negative feedbacks), which must be done on a sufficiently large set in order to yield meaningful results. Actually, the size of the sets used on figures 6 and 7 is already at the very limit.

f) 583
You quite often refer to differences as "more significant" or "very significant". How about using effect size measures to quantify such differences? Please provide effect size measures for your key effects.

We added the effect sizes in the paragraphs devoted to the analysis of the biases in different sets. Moreover, we mentioned the effect size in the section “result treatment”, in the subsection about bootstrap. The changes are reproduced below (in blue):

1000 samples. We considered that this level of precision is sufficient. Bootstrap allows us to compute effect size when comparing the results of a measure on two sets A_1 and

A_2. The effect size is indeed defined as:

$$s = \frac{|m_1 - m_2|}{\sigma_1}, \quad (36)$$

where m_1 and m_2 are the bootstrap averages of the considered measure computed respectively on A_1 and A_2, and σ_1 is the bootstrap standard deviation of the measure for set A_1. An effect size of 0.2 is small, of 0.5 medium, of 0.8 large and of 1.3 very large [42].

The comments of Figures 6 and 7 become the following:

Figure 6 shows that the bias from sensitivity varies around 1% of the feedback intensity. Except for the set of participants of low self-esteem which shows a sensitivity bias close to 0, the bias for other sets varies between 0.73 % (male with evaluation by rank) to 1.34 % of the feedback (male with evaluation by score). For participants of high self esteem and women, the difference between evaluation by rank and by score is

weak (effect size 0.12 and 0.07 respectively). ~~However, the standard deviation from bootstrap samples is in several cases higher than half the value of the mean, indicating a significant uncertainty on the measures.~~ The relative stability of the sensitivity bias is remarkable as the closely associated self-enhancement bias shows ~~significant~~ much stronger variations, as shown on figure 7:

- The self-enhancement bias changes dramatically with the scale: it is significantly positive when participants self-evaluate by rank and significantly negative when they self-evaluate by score in all considered sets (effect size 7.86 between rank and score).
- The self-enhancement bias is higher for men and for participants with high self-esteem than for women and for participants with low self-esteem. The difference is very significant when participants self-evaluate by score (effect size 2.84 between men and women and 3.91 between participants with high self-esteem and women). In the whole data set, the average self-esteem of men (3.08) is only slightly higher than the average self-esteem of women (2.98) and this difference of self-esteem seems insufficient to explain the strong difference of self-enhancement bias between men and women.

g) Citations - I suggest to take APA style but it is up to the editors to decide on that.

We use the Plos latex template. The format can easily be changed anyway.

h) spelling mistakes:
Table 1. heading contains a small mistake ("tow" instead of "two")

Corrected. Thanks.
