# Supplementary material for: A newly detected bias in self-evaluation
Source: PLoS One. 2024 Feb 8;19(2):e0296383. doi: 10.1371/journal.pone.0296383 (PMC10852250; doi:10.1371/journal.pone.0296383)
Supplement: Attachment — Submitted filename: answerToReviewersR5.pdf [file pone.0296383.s017.pdf]

Dear Editor,

We thank you and the reviewer for your feedback on our third revision of the paper.

Please find below our detailed answers to the reviewer's comments (in red).

Sincerely yours,

Guillaume Deffuant

Reviewer #1: As requested by the Academic Editor, we tried to improve our answers to the concerns that reviewer #1 expressed in the previous review.

The first concern of reviewer #1 is about the constraint on the expression of the self-evaluation of the participants. Indeed, the participants should choose their self-evaluation between their last self-evaluation and the feedback. We tried to improve our argument by adding the sentence in blue in the paragraph reproduced below:

- The participants are asked their expected evaluation a_2 in group G_2. They are requested to express this self-evaluation between their previous expectation a_1 and the feedback f_1 that they just received. Doing this, we impose that the sensitivity to the feedback is between 0 and 1 for each observation. The literature (e.g. [31, 32]) and several pilot experiments that we made (not-reported here) suggest that this assumption holds in a large majority of cases. Indeed, participants often put their self-evaluation outside the interval when they do not understand well the requests or do not pay attention. Therefore, the constraint on the self-evaluation is primarily a means to limit the noise in the results. Moreover, the participants are free to choose any value within the bounds. The possible variation of this choice when the self-evaluation varies is thus constrained only in its limits, not in its direction. The main subject of our investigation is precisely the direction of this variation, which we expect to be decreasing. This direction of variation is not constrained by the experimental setting.

Secondly, reviewer #1 requested us to use linear mixed effect models. We think that we completely answered this request, as we applied the method, when applicable, and found results that are very close to the ones obtained with standard regressions.

Reviewer #2: I would like to thank the authors for all the reactions and comments to my suggestions. In general, I think that the responses satisfy my curiosity and adequately address all the caveats I had.

We thank the reviewer for this statement.

Some comments that remain:

a) 109-112:

I think that your discussion of the similarities and differences between your paper and the works of

Gesiarz et al. (2019) and Atir et al. (2015) is very interesting and you should include it somewhere in the paper (maybe in the Discussion section?). One of the main tasks of the authors of any paper, especially one that claims to have found something new, is to comprehensively compare and contrast new findings with what was previously done in the field. It does not suffice to say "it's new", you have to show it. Sometimes it is really new and there are very few studies that directly links to your findings. Then you have to dig somewhat deeper and bring forth also the studies with more indirect links to what you have done.

In my opinion, your discussion does not discuss the many studies indirectly related to your experiments, therefore, I would ask you to improve it by addressing other studies about the interplay of feedback, self-enhancement, self-esteem, etc. This discussion should include studies like Gesiarz et al. (2019) as the task here is also to contrast the present findings with the previous ones and to show, where new knowledge was provided.

I would also like to remind the authors that it is their task to discuss their findings in such a manner to convince the reviewers that the discussion is comprehensive and well-based in the extant literature.

We agree that we should provide convincing arguments for the novelty of our work. However, we have serious difficulties to deal with the suggestion from the reviewer to consider indirectly linked papers and show how our work is different. Indeed, we read with attention the references kindly suggested by the reviewer and all these papers are interesting but they seem very far from our work: none of them considers the sensitivity to feedbacks for instance. Therefore, we do not see why we should cite these papers more than any other paper about bias in self-evaluation.

This is why we privileged a different type of argument for the novelty of our approach. We modified the introduction, trying to make clearer the focus of the paper on a very specific bias and arguing that this specific bias could be detected only in a computer model with perfectly symmetric reactions to positive and negative feedbacks. Indeed, in usual experiments this bias is entangled with self-enhancement or self-derogation bias and cannot be distinguished from them without specifically designed mathematical treatments. This explains why, as far as we know, this bias has not been detected yet and is absent from the social-psychology literature.

We also removed the paragraph of the introduction distinguishing between self-enhancement by impression management from self-enhancement by self-deception and narcissism. Indeed, it seems to us that this paragraph gives too many details about self-enhancement, which is not the subject of the paper. Moreover, the writing of this paragraph confuses the process of self-enhancement and its effect, which is the resulting bias.

We do not reproduce the changes here as they are easily visible in the introduction in the file with marks.

b) 303

OK, now I see what you see and we see it eye to eye. Maybe adding a first column "Serie" or something like that would enhance this table further still, but now I think it is ok. Thank you.

Good.

c) 476

OK, this is rather a semantic discussion – "sample size" is typically used to indicate number of observations, which, in this case, can be either participants or answers. My recommendation is to reframe this section to make it clear what you count as "sample size" in each of the instances/analyses to avoid any confusion between the number of participants and the number of answers collected from them. I also think that the power calculation should be re-run now, to accommodate to the new analytic model employed (hierarchical model with mixed effects).

We tried to clarify that all considered samples include triples (self-evaluation, feedback, next self-evaluation) by the changes reproduced below:

Instead of computing the sensitivities of a single participant, we derive approximations of the sensitivities from ~~data mixing~~ samples of triples $(a_t^i, f_t^i, a_{t+1}^i)$ mixing several participants and several time steps. The larger size of the sample provides higher chances to get significant results. We assume that, in this case, we obtain an approximation of the average sensitivities to feedbacks of the participants in the set.

Hence, considering a ~~set A of participants and time steps~~ sample A of triples $(a_t^i, f_t^i, a_{t+1}^i)$ mixing participants and time steps, we compute the linear regressions taking self-evaluation change $|\frac{a_{t+1}^i - a_t^i}{\delta_t^i}|$ as outcome variable and self-evaluation a_t^i as predictor variable and this provides linear approximations of the sensitivities h, h_p and h_n defined in equations 1, 19 and 20. More precisely, from a given ~~set~~ sample of triples $(a_t^i, f_t^i, a_{t+1}^i)$ we derive the following linear models:

Moreover, we changed "set" into "sample" systematically in this part of the text.

In addition, we realise that the question of the reviewer is more specifically about bootstrap in the case of samples including the four time steps, suggesting that our explanation about this case is not clear. We tried to clarify it. Here is the new paragraph:

Moreover, when the considered ~~subset~~ sample A includes the triples $(a_t^i, f_t^i, a_{t+1}^i)$, for the four time steps $t \in \{1, 2, 3, 4\}$, for each of participant i in set A, then bootstrap helps to evaluate the robustness of the difference between the sensitivity bias (measured as the difference between the total bias and the enhancement bias) and the theoretical sensitivity bias (measured as the average of the sensitivity bias on the sample). If we constitute the samples A^k as usual by randomly choosing triples $(a_t^i, f_t^i, a_{t+1}^i)$, in general, we do not keep the sequences of the four time steps $t \in \{1, 2, 3, 4\}$ complete, thus there is no guarantee to be able to compute the total bias $a_5^i - a_1^i$. Therefore, in this case, instead of deriving the sample A^k by drawing triples $(a_t^i, f_t^i, a_{t+1}^i)$ in A at random, we draw the participants at random (with replacement) and for each drawn participant i, we add the triples $(a_t^i, f_t^i, a_{t+1}^i)$, for the four time steps $t \in \{1, 2, 3, 4\}$ into the new sample A^k.

About the power calculation, the bottleneck remains related to the standard regressions made on positive or negative feedbacks, for which the linear model with mixed effects is not applicable.

d) 553

OK, so be it, please consider adding this point somewhere to the Limitations or Future Directions section then.

We think that this point is rather technical and related to the secondary objectives of the paper. We considered that it is not necessary to mention it in the discussion.
